# Mapping the Melanoma Plasma Proteome (MPP) Using Single-Shot Proteomics Interfaced with the WiMT Database

**DOI:** 10.3390/cancers13246224

**Published:** 2021-12-10

**Authors:** Natália Almeida, Jimmy Rodriguez, Indira Pla Parada, Yasset Perez-Riverol, Nicole Woldmar, Yonghyo Kim, Henriett Oskolas, Lazaro Betancourt, Jeovanis Gil Valdés, K. Barbara Sahlin, Luciana Pizzatti, A. Marcell Szasz, Sarolta Kárpáti, Roger Appelqvist, Johan Malm, Gilberto B. Domont, Fábio C. S. Nogueira, György Marko-Varga, Aniel Sanchez

**Affiliations:** 1 Laboratory of Proteomics/LADETEC, Universidade Federal Do Rio de Janeiro, Rio de Janeiro 21941-598, Brazil; nataliapalmeida@pos.iq.ufrj.br; 2 Proteomics Unit, Institute of Chemistry, Universidade Federal Do Rio de Janeiro, Rio de Janeiro 21941-909, Brazil; gilberto@iq.ufrj.br; 3 Clinical Protein Science & Imaging, Biomedical Center, Department of Biomedical Engineering, Lund University, BMC D13, 22184 Lund, Sweden; nicole.woldmar@pos.iq.ufrj.br (N.W.); barbara.sahlin@med.lu.se (K.B.S.); gyorgy.marko-varga@bme.lth.se (G.M.-V.); 4 Division of Chemistry I, Department of Biochemistry and Biophysics, Karolinska Institute, 17165 Stockholm, Sweden; jimmy.esneider.rodriguez@ki.se; 5 Section for Clinical Chemistry, Department of Translational Medicine, Lund University, Skåne University Hospital Malmö, 20502 Malmö, Sweden; indira.pla_parada@med.lu.se (I.P.P.); johan.malm@med.lu.se (J.M.); 6 European Molecular Biology Laboratory, European Bioinformatics Institute (EMBL-EBI), Wellcome Trust Genome Campus, Hinxton, Cambridge CB10 1SD, UK; yperez@ebi.ac.uk; 7 Laboratory of Molecular Biology and Blood Proteomics—LADETEC, Institute of Chemistry, Federal University of Rio de Janeiro, Rio de Janeiro 21941-598, Brazil; pizzatti@iq.ufrj.br; 8 Data Convergence Drug Research Center, Therapeutics and Biotechnology Division, Korea Research Institute of Chemical Technology (KRICT), Daejeon 34114, Korea; yonghyo@krict.re.kr; 9 Division of Oncology, Department of Clinical Sciences Lund, Lund University, 22185 Lund, Sweden; henriett.kovacsne_oskolas@med.lu.se (H.O.); lazaro_hiram.betancourt_nunez@med.lu.se (L.B.); jeovanis.gil_valdes@med.lu.se (J.G.V.); roger.appelqvist@bme.lth.se (R.A.); 10 Oncology Center, Semmelweis University, 1083 Budapest, Hungary; szasz.attila_marcell@med.semmelweis-univ.hu; 11 Department of Dermatology, Venereology and Dermatooncology, Semmelweis University, 1085 Budapest, Hungary; karpati.sarolta@medsemmelweis-univ.hu; 12 Chemical Genomics Global Research Lab, Department of Biotechnology, College of Life Science and Biotechnology, Yonsei University, Seoul 03722, Korea; 13 Department of Surgery, Tokyo Medical University, 6-7-1 Nishishinjiku Shinjiku-ku, Tokyo 160-0023, Japan

**Keywords:** malignant melanoma, plasma, proteome, proteomics, biomarkers, WiMT

## Abstract

**Simple Summary:**

We developed a clinical proteomics methodology, known as Wise MS Transfer (WiMT), for deep identification of blood proteins in undepleted plasma samples. We applied it to the analysis of undepleted melanoma plasma samples as a proof of principle. Malignant melanoma is the most aggressive type of skin cancer, and early diagnostic and prognostic predictors are essential to establish the most suitable treatment tailored to the patient. Our results showed the greatest identification of proteins and biological processes to date reported for a “dilute and shoot” approach within plasma samples from melanoma patients. More than 1200 proteins related to key biological processes in melanoma progression were mapped, including signaling (the PI3K–Akt signaling pathway), immune system processes (complement and coagulation cascade), and secretion (exosome proteins). These proteins and related biological processes constitute the core of blood components that could be monitored by mass spectrometry in clinical proteomic studies from undepleted plasma samples in melanoma.

**Abstract:**

Plasma analysis by mass spectrometry-based proteomics remains a challenge due to its large dynamic range of 10 orders in magnitude. We created a methodology for protein identification known as Wise MS Transfer (WiMT). Melanoma plasma samples from biobank archives were directly analyzed using simple sample preparation. WiMT is based on MS1 features between several MS runs together with custom protein databases for ID generation. This entails a multi-level dynamic protein database with different immunodepletion strategies by applying single-shot proteomics. The highest number of melanoma plasma proteins from undepleted and unfractionated plasma was reported, mapping >1200 proteins from >10,000 protein sequences with confirmed significance scoring. Of these, more than 660 proteins were annotated by WiMT from the resulting ~5800 protein sequences. We could verify 4000 proteins by MS1t analysis from HeLA extracts. The WiMT platform provided an output in which 12 previously well-known candidate markers were identified. We also identified low-abundant proteins with functions related to (i) cell signaling, (ii) immune system regulators, and (iii) proteins regulating folding, sorting, and degradation, as well as (iv) vesicular transport proteins. WiMT holds the potential for use in large-scale screening studies with simple sample preparation, and can lead to the discovery of novel proteins with key melanoma disease functions.

## 1. Introduction

The diagnosis and prognosis of malignant melanoma (MM) is mainly determined by histological tumor characterization and by its staging [1]. There is, however, an increasing need to identify predictive molecular biomarkers serologically, as blood samples can be obtained in a minimally invasive manner [2,3]. Although plasma holds most of the blood components, the characterization of plasma/serum proteomes is still challenging, especially for low-abundant protein expression. Covering the entire proteome is difficult due to its large dynamic range (which is more than 10 orders in magnitude) and the presence of a small group of proteins in high concentrations (such as immunoglobulins and albumin) which represent 99% of the total plasma protein content [4,5,6].

In the 2000s, the search for new MM biomarkers was performed mainly by proteomic fingerprinting and two-dimensional gel electrophoresis coupled to mass spectrometry (MS) analysis, with the identification of only a few proteins or proteomic profiles that could distinguish patient groups from different disease stages [7,8,9]. Immunodepletion of the most abundant proteins [10], sample fractionation, or a combination of these methodologies can allow for a deeper characterization of plasma/serum MM proteomes. This improves the number of identifications from a few hundred to thousands of proteins by LC-MS/MS [11,12,13,14,15,16].

Currently, there is a lack of techniques and methodologies able to encompass the entire plasma/serum proteome without modifying sample characteristics. This is essential for accurate protein quantification in clinical proteomic studies [17,18,19].

Different strategies have successfully been adopted by MS-based proteomic workflows for the characterization of low-abundant proteins in samples with large dynamic ranges. The procedures explore and/or improve either MS1 or MS2 events by MS in combination with a centered-designed database to reduce the search space [20,21,22,23,24,25,26,27]. At the MS1 level, these strategies focus on MS1 information transference (MS1 transfer or MS1t) between experiments. This means that the identification of peptides achieved by comparing the eluting precursors in different chromatographic runs with high mass accuracy and reproducible retention times is ensured for correct assignments [18,19,28,29,30,31,32]. The MS1t principle has been reflected in many practical applications, such as the match between runs in the MaxQuant software (MBR) [32,33,34,35], or has been simply defined as the transfer of MS1 features with easy-interfaced software/algorithms such as OpenMS [34,36,37,38] or Proteome Discoverer [39] (Thermo Fisher Scientific, San José, CA, USA) to increase identifications and to improve label-free quantification (LFQ) workflows by reducing missing values. In 2016 and 2019, Geyer et al. [18,19] applied MBR software to increase the plasma proteome coverage by transferring MS1 information. This was completed using a database of depleted and fractionated plasma samples without previous functional characterization of the proteins identified in the database. This strategy allowed the identification of around 1000 proteins, reaching the low-abundance region down to ~10 ng/mL.

In the present work, we describe the melanoma plasma proteome (MPP) obtained from the analysis of undepleted and unfractionated plasma samples from patients with malignant melanoma. This is performed using the Wise MS Transfer procedure (WiMT), which includes the MS1t principle, a custom in-depth database, and single-shot proteomics. The custom database was built and characterized using different immunodepletion strategies for plasma samples. The information covered by the database was transferred to plasma samples from MM patients by MS1t. We suggest increasing the undepleted MPP coverage by performing a single nLC-MS/MS run (using a nano liquid chromatography system interfaced to high-resolution mass spectrometry) without any prior interference with the sample integrity. The database design is simple and tracks protein abundance according to the depletion level required for positive annotation and identification. Most of the proteins from the database have been identified in the unfractionated samples, allowing for deeper characterization of undepleted MPP, in which the main biological processes and protein classes were successfully mapped.

## 2. Results and Discussion

### 2.1. Custom Database Development

**Figure 1:**
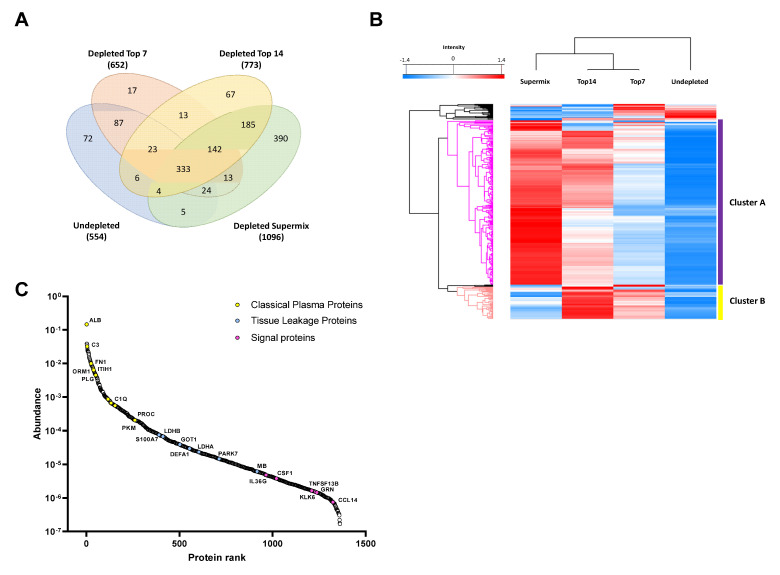
Descriptive results of the proteomic analysis of immunodepleted plasma samples. (**A**) Comparative analysis of the number of proteins identified by each approach. (**B**) A hierarchical clustering heat map of the proteins identified as common among the 4 groups of samples studied. The gradient from blue to red represents the Z-score scale ranging from −1.4 to 1.4. (**C**) Protein abundance distribution curve. Classical plasma proteins, tissue leakage, and signaling proteins are highlighted. Tissue leakage proteins were defined as plasma proteins that are not secreted into the blood stream, classified as intracellular proteins (by available information) according to The Human Protein Atlas database (https://www.proteinatlas.org/search/protein_class:Plasma+proteins, and https://www.proteinatlas.org/humanproteome/blood+protein/secreted+to+blood, accessed on: 5 March 2020) [45,46,47].

We developed a custom database containing more than 1300 proteins identified in plasma from malignant melanoma patients using different immunodepletion approaches (Table S1). The proteins were categorized depending on the strategy applied and we related the protein abundances with the depletion level needed for identifying the proteins in the database. The custom database was divided into four levels depending on the degree of plasma depletion. The undepleted samples represent the first level, and the top7, top14, and SuperMix strategies constitute the second (Low-Dep), third (Mid-Dep), and fourth levels (Deep-Dep), respectively. The database contains a total of 1385 identified proteins, of which 554 are from undepleted plasma, and with the immunodepletion strategies the number of identified proteins increased by ~18%, 40%, and 98% for top7 depletion, top14, and SuperMix, respectively (Figure 1A). While many reports have compared the immunodepletion strategies in terms of efficiency, reproducibility, and specificity [16,40,41,42], to the best of our knowledge this study is the first to surpass 1000 identified proteins without previous sample fractionation, using a simple “dilute-and-shoot” approach. Unsupervised hierarchical clustering of quantified proteins showed that depleted samples clustered together (Figure 1B). Thus, it was possible to observe the quantitative difference between the immunodepletion approaches, confirming the enrichment of lower abundant proteins (Cluster A). Cluster B comprises those proteins that are enriched in Low-Dep and Mid-Dep levels but show a decrease in abundance at the Deep-Dep level. As previously stated, the SuperMix depletes 50–60 highly abundant proteins from plasma [10,43,44]. The proteins identified in cluster B and the proteins reported to be captured using SuperMix sample preparation have been compared [43]. At least 52% of the proteins within the cluster were depleted by this methodology (Table S2). Figure 1A shows the numbers of unique proteins identified in undepleted and depleted samples. A comparison of the top7 and top14 strategies with the undepleted plasma results showed that most of the proteins lost in the process were immunoglobulins, at ~32%, and 53%, respectively. Excluding the immunoglobulins, approximately 83% of the proteins were lost due to top7 depletion. Consequently, these were also lacking when applying the top14 strategy. The protein abundance distribution is illustrated in Figure 1C. Data collection from undepleted and depleted samples using different strategies can compensate for this loss of information, allowing a broader coverage of the plasma proteome.

### 2.2. Functional Building and Characterization of the Custom Database

We were able to characterize the custom protein database according to:

- biological processes;
- protein classes; and
- pathway biology and enrichment within these signaling cascades.

We were able to verify the functional correlations by applying the respective depletion levels. Protein enrichment is directly related to the increase in plasma proteome coverage. Proteins considered as classical plasma proteins, proteins deriving from tissue leakage, and signaling proteins were identified (see Figure 1C). Most of the tissue leakage proteins were concentrated in the region of the medium abundance of the plasma proteome, as has been previously discussed [2,4]. Signaling proteins such as interleukin-36 gamma (IL36G), macrophage colony-stimulating factor 1 (CSF1), tumor necrosis factor ligand superfamily member 13B (TNFSF13B), and C-C motif chemokine 14 (CCL14) were successfully identified. It was possible to access larger ranges of concentration in the plasma proteome since the number of low abundance proteins identified increased from the Low-dep to Deep-dep level. For instance, we were able to identify 141, 301, 601, and 62 proteins at a concentration level of <100 µg/L (low-abundance proteins, LAP) [48], according to the Human Protein Atlas (https://www.proteinatlas.org/humanproteome/blood+protein, accessed on: 5 March 2020), using the top7, top14, SuperMix, and undepleted plasma approaches, respectively [45,46,47].

**Figure 2:**
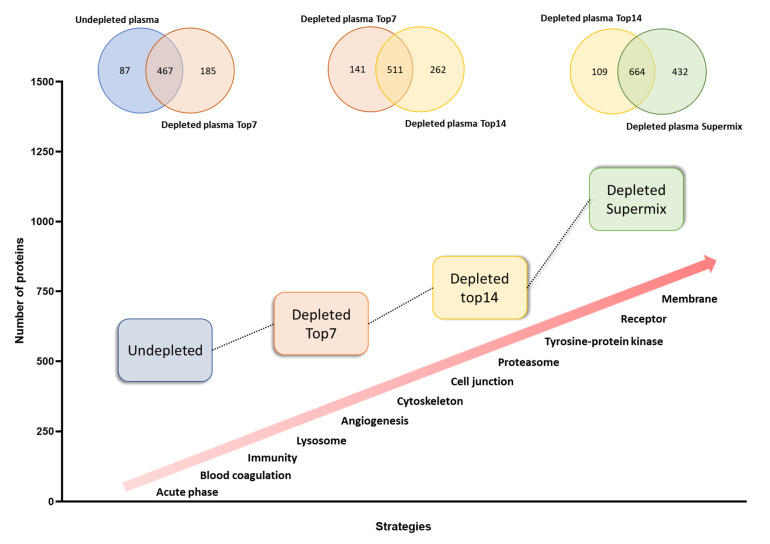
Qualitative evaluation of whole and immunodepleted plasma proteomes. Characterization of the 4 strategies used in this work according to the number of proteins identified and the specifically enriched protein classes or biological process.

Proteins identified at each depletion level were submitted to functional annotation enrichment analysis. As expected, proteins related to the acute phase, blood coagulation, and complement pathway were not enriched by any of the immunodepletion techniques (see Figure 2 and Table S3), since most of these proteins can be found in the high and medium concentration ranges [18]. The Low-dep strategy improved the identification of proteins related to angiogenesis, lysosomes, and cell projection, as well as the cytoskeleton. Although there was a higher number of proteins identified with the Mid-dep approach as compared with the Low-dep approach, it was possible to see similarities between the two methodologies, particularly for signaling and secreted proteins. For both strategies, most of the proteins lost in the process were immunoglobulins. The Mid-dep strategy improved our identification, with the enrichment of cell junction, proteasome, tyrosine kinase, and stress response protein kinases. Remarkably, we could annotate membrane, transmembrane, and receptor proteins, together with tissue remodeling and MHC I proteins enriched using the Deep-dep approach.

**Figure 3:**
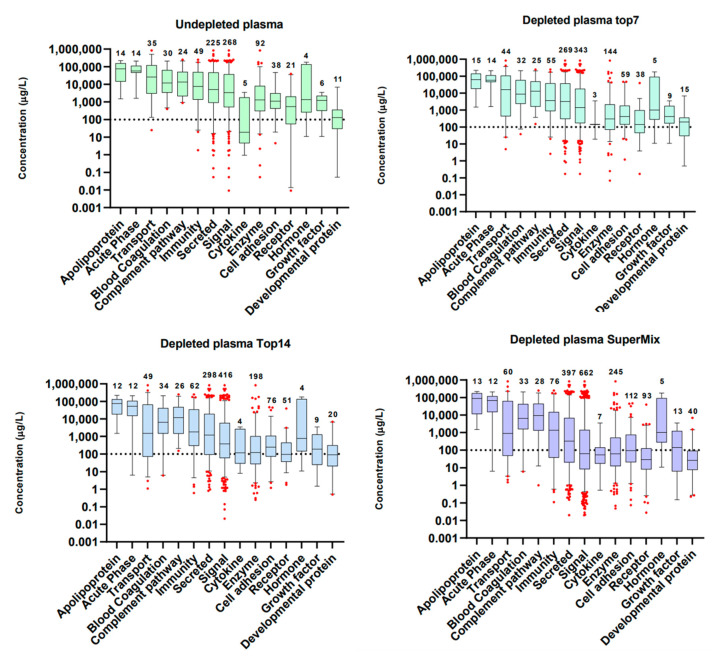
Functional groups identified in whole and immunodepleted plasma samples. The proteins were annotated using the information included in The Human Protein Atlas database for plasma proteins (https://www.proteinatlas.org/search/protein_class:Plasma+proteins, accessed on: 29 July 2020) [45,46,47]. The graphs were built using the protein concentrations in blood reported in the same database. The boxes represent the median and whisker ranges: 5th–95th percentiles.

The results showed a close association between the depletion level and the enrichment of groups of proteins with related functions. As a general trend, the deeper the immunodepletion approach, the higher the number of proteins identified per functional groups and at lower concentrations (Figure 3). Our approach allows the identifications of growth factors, which are known to be present in plasma in concentrations around ng/L, as described in The Human Blood Atlas (Available in: https://www.proteinatlas.org/humanproteome/blood+protein, accessed on: 11 August 2020) [45,46,47]. These proteins are commonly identified by immunoassays, whereas the detection by MS still is a challenge [49]. This means that the customized database has great potential for biomarker research [2]. To verify the validity of the WiMT developments, known and established melanoma biomarkers are clearly identified, including lactate dehydrogenase, metalloproteinases, and S100 proteins [50].

We also found the enrichment of proteins related to the biosynthesis of amino acids, carbon metabolism, and glutathione metabolism pathways (Table S4), which have been related to different disorders such as cancer and neurodegenerative diseases [51,52,53,54,55,56,57]. The PI3K–Akt signaling pathway has been found to be altered in several types of cancer, including melanoma. It regulates multiple (patho)physiological processes such as cellular growth, survival, invasion, and angiogenesis in melanoma [58]. This pathway is enriched at the Mid-dep and Deep-dep levels, showing a higher number of proteins identified in the latter. Therefore, we can achieve a better understanding of some aspects of these diseases and discover potential biomarkers.

Other strategies can be applied to improve database development and consequently peptide/protein identification by WiMT, including extensive fractionation of immunodepleted samples and/or the addition of orthogonal enrichment methods such as ProteoMiner^®^ [59]. However, the inclusion of fractionated samples requires an improvement in bioinformatic strategies for chromatogram alignment and MS1 transfer.

Since the database was mainly built based on samples from healthy individuals, its applicability is not restricted to melanoma studies, but could be applied to other diseases. We included the undepleted data from the analysis of a plasma pool from melanoma patients to maintain the main characteristics of melanoma to the greatest degree possible. Consequently, this enabled us to identify low-abundant proteins that could not be identified in the analysis of a single sample. More specific proteins could be identified by developing a personalized database with depleted samples from patients with the disease in question (in our case melanoma patients). In WiMT, the researcher can adapt the library to respond the biological question.

### 2.3. Evaluating the MS1 Transfer Procedure (MS1t)

We optimized an experimental model using diluted HeLa protein digests to evaluate the MS1t. The Hela digest is a well-known standard and is commonly used by the proteomics community to evaluate the performance of instruments, new sample preparations, or data acquisition methodologies [27,60,61]. It has also been applied in the evaluation of other MS1 transfer methodologies and single-cell proteomics [62,63]. In addition, most of the proteins identified in different types of cancer cell lines can be found in Hela, which means that this standard provides a qualitative representation of different cell proteomes [64].

The MS1t consists of the transference of MS1 features between two sets of MS data for the identification of peptides, ensuring high mass accuracy and reproducible retention times for the comparison of the eluting precursors [18,19,28,29,30,31,32]. However, these biological fluids contain proteins at a high dynamic range concentration, and in some cases the input of material to be analyzed by LC-MS/MS may be limited. In fact, these factors could compromise MS1t efficiency with regard to both quantitative and qualitative aspects. In this context, HeLa digest dilutions series were utilized to evaluate the dynamic and linearity ranges of MS1t.

**Figure 4:**
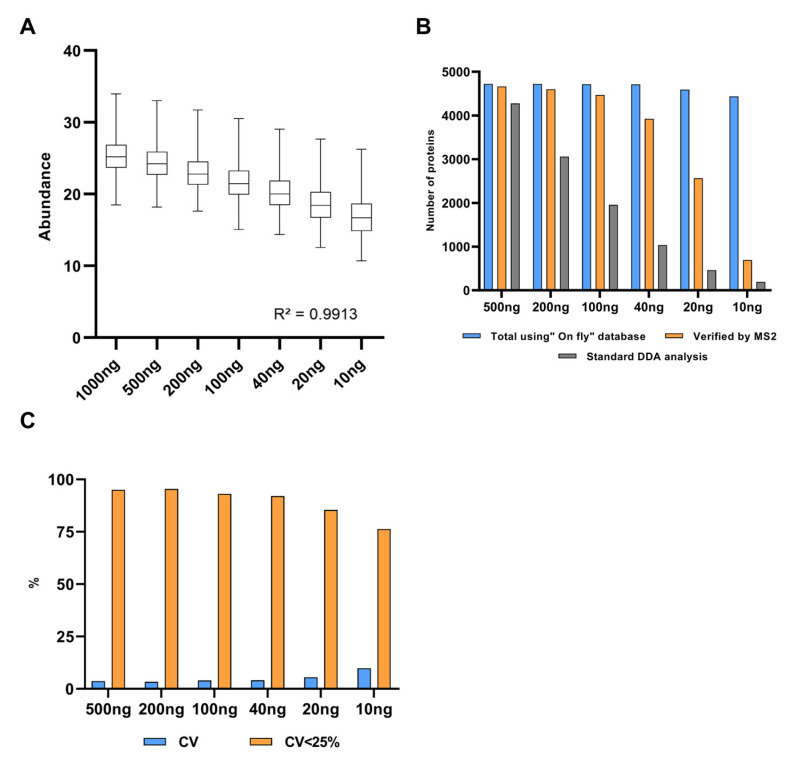
A HeLa experimental model for MS1 feature transfer evaluation. (**A**) Linearity analysis of protein abundance depending on the protein amount analyzed. Abundance: Log2 (intensity). (**B**) Comparison of the results obtained by MS1t, DDA, and DIA analyses (verified by MS2). (**C**) MS1t coefficient of variation evaluation throughout the dilution points. Blue bars represent the median CV values of dilution points, and orange bars show the percentages of proteins with cv lower than 25%.

Overall, the MS1t analysis allowed us to verify approximately 4000 protein identifications linearly transferred from 1 μg to 10 ng analyses. Figure 4A confirms that the regression coefficient using the protein intensity medians per concentration (or dilution) group was greater than 0.99. This demonstrates that MS1t maintains linearity, and thus the input material is reduced. To assess the identification data in detail, comparisons between MS1-t, standard DDA, and DIA analysis for each dilution were proven (see Figure 4B). As expected, the identification rate decreased dramatically as the input material was lowered in DDA mode. At 200 ng, the difference between MS1t and DDA was about 1500 proteins, and with 10 ng, this difference increased to more than 3500 proteins. In the same way, in DIA mode (MS2 acquisition) the protein identification decreased dramatically in samples with the lowest input material (<40 ng). In contrast to standard DDA and DIA, MS1-t appeared consistent across the dilution setting. This was because the intensity of some precursors at MS1 was not high enough to select those precursors for fragmentation in DDA. We observed that in DIA experiments, the low intensity of fragments could be the reason for the lack of a positive identification. Thus, MS1-t takes advantage of MS1 detection features to increase proteome coverage. On the other hand, MS1t appeared robust in terms of variation, as shown in Figure 4C, where the median CV values of all dilution points remained lower than 10%. We found that more than 75% of the proteins in all cases had a CV < 25%.

The previous MS1t analysis was also performed in grouped proteins according to their abundance. In all dilution points, proteins identified by MS1t and DDA were ranked by their respective intensities and divided into 10 groups of ~300 proteins in each. As illustrated in Figure S1, linearity was maintained across the groups regardless of the protein abundance, ensuring that MS1t was achievable in proteins with at least 2–3 orders of linearity. When MS1t was contrasted with DDA and DIA in the different groups, it was evident that MS1t became significant as the protein abundances decreased, especially at low levels (Figure S2). Particularly, with 40 ng or less input material, low-abundance proteins were accessible mostly by MS1t (groups 8, 9, and 10). Overall, these results indicate that proteins present in the nanogram range could be accessible “exclusively” by MS1t with high transference confidence. This is particularly relevant in plasma/serum studies where the proteome covering could be improved by applying the MS1t concept, especially for low-abundance key regulators. Furthermore, the HeLa model was successfully applied in the evaluation of our methodology; however, use of a melanoma cell line could reveal additional information regarding key and/or low-abundance melanoma proteins for their effective identification through MS1 transfer.

### 2.4. MM Plasma Proteome Assessment by Applying WiMT

Unlike other methodologies for MS1 transferring in plasma that used extensive depletion top 14–20 together with previous peptide fractionations [18,19] we developed a strategy to use single-shot proteomics (without peptide fractionation), taking advantage of the power of a SuperMix depletion (reaching more than 1000 proteins after LC-MS) combined with other depletion strategies in order to complement the specific losses during each stage of depletion. Thus, in a single-shot experiment it is possible to run each depletion method using the same LC-MS condition in which the undepleted samples are analysed, while keeping the MS1 transfer as simple as possible and in an equivalent way to how it was done with diluted Hela.

The custom database described in the first section was applied for peptide identification using MS1t from immunodepleted to undepleted plasma from MM patients, with more than 1200 proteins and 10,000 peptides identified in total with significant scoring. About 660 proteins and ~5800 peptides were annotated by WiMT (Tables S5 and S6). Although the presence of these peptides was not inferred by MS2 spectra annotation, their presence was confirmed by MS1t transfer evaluation and FDR filtering since we could provide evidence of a great improvement in protein identification using a multiple dilution strategy with the HeLa experiment. Furthermore, 80% of the proteins reported on The Human Melanoma Proteome Atlas from depleted plasma samples of the same patients were also identified here [65]. Additionally, our improvement in protein identification is like that reported by Geyer et al. in 2016 [18].

**Figure 5:**
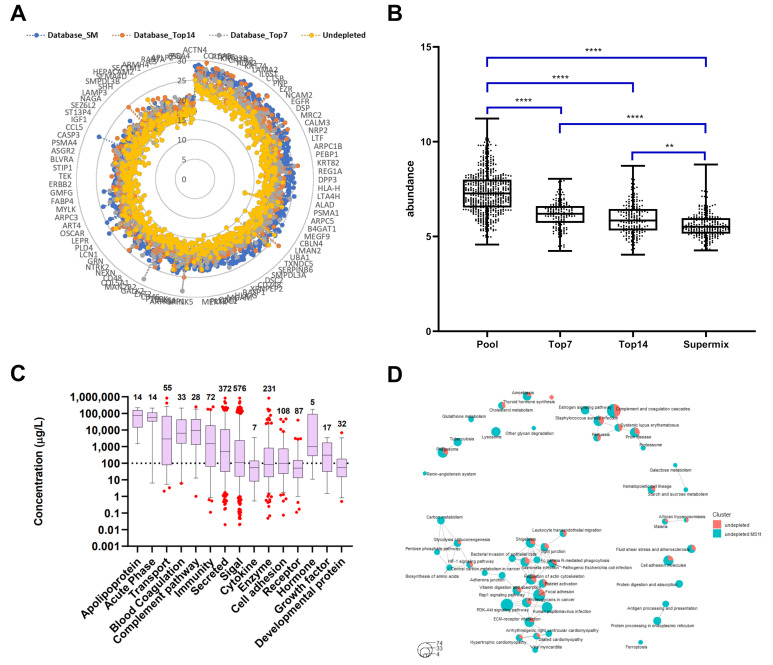
Assessment of proteins identified in undepleted plasma by WiMT. (**A**) A graphic representation of the 4-layer custom database. The layers represent the depletion levels and the intensities of the proteins are represented by colors. (**B**) Protein classification according to their depletion levels. The y axis refers to the protein abundance in undepleted plasma samples. One-way ANOVA test (GraphPad Prism 8.3.1): ** <0.01; **** >0.0001. (**C**) Proteome profiling of undepleted plasma applying MS1t for peptide identification. The proteins were annotated using the information included in The Human Protein Atlas database for plasma proteins (https://www.proteinatlas.org/search/protein_class:Plasma+proteins, accessed on: 29 July 2020) [45,46,47]. The graphs were built using the protein concentrations in blood reported in the same database. The boxes represent the median and whiskers for the 5th and 95th percentiles, respectively. (**D**) KEGG pathway enrichment analysis for the comparison of undepleted plasma before and after MS1t. Each circle represents a pathway, while the size of each circle is related to the number of proteins, and the colors differ from the results obtained before and after MS1t.

The total expression dataset analyzed from melanoma patients is important as it builds on the expansion of our melanoma database over time. The WiMT approach increased the number of proteins identified in undepleted plasma and consequently enriched several biological processes and pathways that could only be accessed at the highest levels of depletion. The database was built with a depletion protocol, without using any sample fractionation procedures. Therefore, each depletion method is represented by nLC-MS/MS shotgun sequencing, providing the MS1 features and enabling good chromatogram alignment for the MS1t. A graphical representation of the four-layer custom database is shown in Figure 5A. The intensities of the proteins transferred to the undepleted samples are represented by colors depending on the depletion level. The proteins identified show different intensities according to the immunodepletion strategy applied due to MS signal improvements. Thus, higher-intensity layers are associated with the plasma depletion extension (Low-dep, Mid-dep, and Deep-dep). With this strategy, 1088 proteins were identified in a pool of undepleted plasma samples from MM patients (Table S7). Approximately 81–94% of the proteins identified in each of the 3 immunodepletion strategies were transferred to the undepleted plasma samples. As discussed in The Human Melanoma Proteome Atlas [65], we could also identify more than 60% of the FDA-approved biomarkers. The identification in the non-depleted samples of more than 80% of the proteins identified from this pool of metastatic depleted melanoma samples supported the identification process [65]. The analysis included 12 potential MM biomarker candidates such as lactate dehydrogenase, C-reactive protein, serum amyloid A, osteopontin, and the melanoma cell adhesion molecule, among others (Table 1). In general, the deregulation of these protein abundances is also associated with other types of cancers and even with other diseases, which means that these are not melanoma-specific [66,67,68,69,70,71,72]. However, their abundances can be included in protein signatures/patterns to associate changes related to the physiological characteristics of melanoma patients.

In addition, the WiMT strategy covered more than 40% (176/435) of the secreted blood proteins previously identified by immunoassays and collected based on published research articles as described in The Human Protein Atlas (https://www.proteinatlas.org/humanproteome/blood+protein/proteins+detected+by+immunoassay, accessed on: 28 November 2021) [45,46,47]. There are 55 out of 110 currently FDA-approved blood biomarkers in the list above, with just 11 falling within the very- to ultra-low abundance protein range, i.e., from 10 µg/L to lower 10 ng/L, respectively. In this context, none of these proteins were identified by applying WiMT, nor have they ever been identified in similar studies on undepleted plasma [18].

However, the WiMT strategy allowed the identification of 247 proteins with concentrations under 10 ng/mL, which opens up the possibility of identifying novel melanoma biomarkers with this approach, as has been predicted when more than 1000 proteins are identified in plasma samples [2].

To support the identification process, the multi-level design of the database allows us to categorize the proteins identified in the undepleted samples according to the depletion levels information that we obtained from the well-characterized database. Significant differences were observed in protein abundances when comparing the four levels (Figure 5B). A decrease in the median abundance of proteins categorized as identified in undepleted to SuperMix was observed. These results support the identification process based on a clear association between the protein expressions in the undepleted samples and the depletion levels from the database. No significant difference was found between the top7 and top14 categories, as expected from the characterization of Low-Dep and Mid-Dep levels.

Although the number of proteins and their intensities from top7 to top14 increases, this increment is not sufficient for the observation of statistically significant changes. More importantly, this shows that it is possible to increase the coverage of the MPP using a WiMT strategy similar to the one achieved with the database. Furthermore, the identification of the same functional groups enriched by these immunodepletion approaches (See Figure 3 and Figure 5C) was achieved. The increased proteome coverage was reached without any additional steps in sample processing. In this way, the sample quantitative characteristic was maintained, helping to ensure protein transfer.

Figure 5D shows the pathways enriched in the analysis of non-depleted plasma as compared with the results obtained with WiMT. This resulted in the enrichment of the pathways discussed above, including the PI3K–Akt signaling pathway, the biosynthesis of amino acids, carbon metabolism, adherens junctions, proteasomes, and lysosomes. In addition, the WiMT increased the number of proteins identified in relation to some of these pathways.

**Figure 6:**
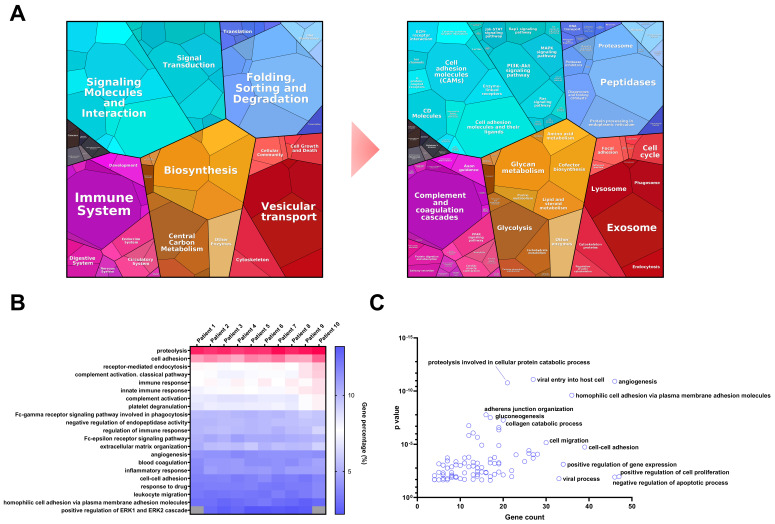
Plasma proteome characterization of MM patients. (**A**) Plasma proteomap based on KEGG pathway enrichment. The analysis covered 57.8% of the MM plasma proteome. The results are grouped into 6 main groups. Light blue: Environmental information processing. Dark blue: Genetic information processing. Pink: Organismal system. Orange: Metabolism. Red: Cellular processes. Black: Human diseases. (**B**) Comparative analysis of the biological processes identified in the undepleted plasma of 10 MM patients using a custom database and MS1t strategy. This analysis was performed using the DAVID functional annotation tool, considering the results with *p*-value and FDR < 0.05. (**C**) Biological processes specifically enriched in 10 MM patients using MS1t (at least 50% of the patients). This analysis was performed using the DAVID functional annotation tool, considering the results with *p*-value and FDR < 0.05.

In addition, WiMT was applied to the analysis of plasma samples from 10 malignant melanoma patients in the early stages of the disease, i.e., when the primary tumors were detected. Using the custom database, 1134 proteins were identified (Table S7). Notably, almost 90% of the proteins identified in a pool of depleted samples from metastatic melanoma patients [65], including the potential melanoma biomarkers, were covered using the WiMT methodology in undepleted samples. Most of the biomarkers (Table 1) can be found in all the patients, except for osteopontin, which was identified in 40% of the samples. The proteomap based on KEGG pathway enrichment showed that the plasma proteome can be divided into six major groups: environmental information processing, genetic information processing, organismal systems, metabolism, cellular processes, and human diseases (see Figure 6A). These groups can be further divided into categories such as signaling molecules and interaction, signal transduction, biosynthesis, the immune system, and vesicular transport, for instance. The third level of categorization shows the detection of proteins related to the PI3K–Akt, MAPK, and Ras signaling pathways, cell adhesion molecules, complement and coagulation cascades, glycolysis, peptidases, proteasomes, lysosomes, and exosome proteins. Gene ontology analysis showed that more than 200 biological processes were enriched, including the immune system, cell adhesion, angiogenesis, inflammatory response, and positive regulation of the ERK1 and ERK2 cascade. These biological processes and pathways have been found to be dysregulated in many types of cancers, including melanoma, which indicates that the proteins identified could be potential biomarkers, as discussed previously. Evaluating the percentages of genes identified in each process, the 10 patients showed similar results, indicating good reproducibility of the results among the patients using WiMT strategy (see Figure 6B). When comparing the biological processes enriched using the MS1t strategy and the protein identification by MS2 spectra annotation only, 102 biological processes were specifically enriched. Within these, angiogenesis, cell adhesion, adherens junction organization, cell migration, and the regulation of cell proliferation were identified as constituting the main biological processes (Figure 6C).

Quantitative results showed a good correlation between the two MM pool replicates, with R = 0.97 and a *p*-value < 0.0001 (Pearson correlation) (Figure S3), and a coefficient variation lower than 10% (85% of the proteins with CV 25%). Similar results were obtained in other experiments with undepleted plasma samples using the WiMT strategy, with a CV < 10% and Pearson correlation above 0.96 (*p*-value < 0.0001). These results showed a good correlation (R^2^ of 0.6154) between the estimated protein abundances by mass spectrometry and the estimated protein concentrations in blood (available in: https://www.proteinatlas.org/humanproteome/blood+protein, accessed on: 5 March 2020) [45,46,47], which were to be compared to the values obtained previously [18] in the analysis of undepleted plasma with similar strategies (See Figure S3). A good correlation was also observed as a resulting outcome from the 10 MM patients, with a mean R = 0.92 (Pearson correlation) and a coefficient variation lower than 5% (Figure S4).

Altogether, our results provide evidence of a successful MS1 transfer, generating the identity and quantification of more than 1200 proteins in undepleted plasma and detecting 12 out of 17 potential MM biomarker candidates. Here, the WiMT was applied to characterize MPP; however, this strategy can be expanded for the study of different pathologies. Although the WiMT is based on well-known concepts (MS1 transfer), the accuracy and methods for controlling the false discovery rates after transfer depend on the platforms used and have previously been discussed [29,31,33,36,78,79]. For reliable MS1 transfer, a highly reproducible retention time and accurate determination of *m*/*z* are required for chromatogram alignment. Therefore, the use of robust HPLC systems coupled to high-resolution mass spectrometry such as Q Exactive HF-X (ThermoScientific) is indispensable. We strongly recommend the use of WiMT only for relative quantification in discovery proteomics, and complementing the analysis, when possible, with orthogonal experiments and biological or clinical information. The validation of selected differentially expressed proteins could be performed in another cohort by use of low-resolution mass spectrometers such as triple quadrupoles, or immunoassays.

## 3. Materials and Methods

**Figure 7:**
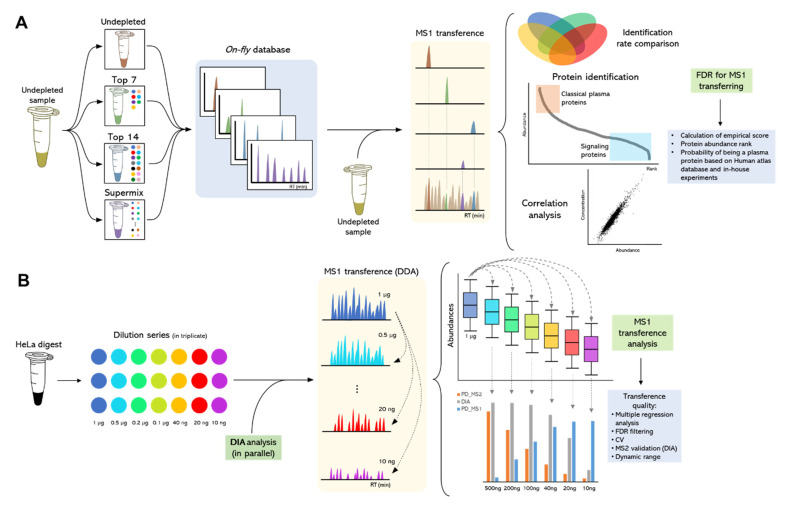
Experimental WiMT workflow for the assessment of low-abundance proteins in undepleted plasma samples. (**A**) The strategy used for the development of a custom database and its application for peptide identification using MS1t. (**B**) Experimental model design for the evaluation of MS1t strategy using a 6-time diluted commercial HeLa sample.

Figure 7 depicts the WiMT developed and applied in the present study. The approach is divided into 3 steps: (1) the development and characterization of a 4-layer custom database by using 3 plasma immunodepletion strategies and nLC-MS/MS; (2) evaluation of MS1t efficiency throughout the analysis of a series of diluted HeLa samples; and (3) A custom database applied for the plasma proteome assessment of MM patients.

### 3.1. Blood Sample Collection and Storage

Blood sample collection was performed before tumor resection surgery at Semmelweis University Hospital. The samples underwent automated fractionation into plasma, serum, lymphocytes, and erythrocytes [80,81] and were stored at −80 °C within 2 h. The samples were then transferred in dry ice to the melanoma biobank (Lund, Sweden) where they were stored at −80 °C until further processing. The project was approved by the local Ethical Committee 727 and the Ethical Committee at Semmelweis University (191-4/2014), as well as the Swedish Ethical Review Authority in Lund (code DNR 2014/311). All patients provided written informed consent. Here, the analyses were performed using a pool of 57 MM patients at different stages of the disease, plasma samples from 10 patients at the primary tumor stage, and a pool of 30 healthy individuals.

### 3.2. Development of a Custom Database for MS1-Transferring

#### 3.2.1. Plasma Immunodepletion

A pool of plasma samples from healthy individuals (*n* = 30) was depleted using a Multiaffinity Removal Column human-7 (4.6 × 50 mm), Multiaffinity Removal Column human-14 (4.6 × 100 mm) (Agilent Technologies, Santa Clara, CA, USA), and Seppro^®^ SuperMix LC2 (6.4 × 63 mm) (Sigma-Aldrich, St. Louis, MO, USA) coupled to a 1260 Infinity LC System (Agilent Technologies, Santa Clara, CA, USA). Each immunodepletion protocol was performed according to the manufacturer’s instructions on technical replicates (Figure S5A–C). To eliminate the variation caused by technical issues, the replicates for each strategy were pooled together for further steps.

After depletion, the samples were submitted to a buffer exchange using an Amicon Ultra Centrifugal filter (0.5 mL–10 kDa, Millipore, County Cork, Ireland). Briefly, samples were transferred to the Amicon 10 kDa and centrifuged at 13,000× *g* for 20 min. Then, 400 µL of 50 mM ammonium bicarbonate (Ambic) was added, followed by centrifugation at 13,000× *g* for 20 min. This step was repeated, and centrifugation was carried out for 30 min. Lastly, 70 µL of 10% of sodium dodecyl sulfate (SDS)/25 mM of 1,4-dithiothreitol (DTT) in 100 mM of triethylammonium bicarbonate buffer (TEAB) were added, the Amicon was turned upside down, and the sample was recovered in a tube by centrifugation at 1000× *g* for 5 min.

#### 3.2.2. Samples Digestion

Samples were digested in an S-Trap (Protifi, Farmingdale, NY, USA) plate, as described by Kuras et.al in 2020 [82]. Briefly, 70 µg of protein, quantified by Pierce 660 nm protein assay (Thermo Scientific, Waltham, MA, USA), was used for sample processing in the top7 approach, and all protein content in top14 and SuperMix. For sample reduction, samples were incubated in SDS/25 mM DTT in 100 mM TEAB for 5 min at 99 °C, with shaking at 500 rpm. Alkylation was performed with iodoacetamide with a final concentration of 50 mM for 30 min at room temperature in the dark. The samples were then acidified by adding orthophosphoric acid to a final concentration of 1.2% and diluted 7× with binding buffer (90% methanol, 100 mM TEAB). Samples were transferred to the S-Trap plate and captured proteins were washed 4 times with a binding buffer. Each step was performed with centrifugation at 1000× *g* for 2 min. The protein digestion was carried out by adding LysC (Wako Chemicals, Richmond, VA, USA) in 50 mM TEAB in a ratio of 1:50 (enzyme: protein) and incubating the S-trap plate at 37 °C for 2 h, followed by the addition of trypsin (Promega, Madison, WI, USA) (1:50) in 50 mM TEAB and incubation at 37 °C overnight. Peptide elution was performed in 3 steps by adding 80 µL of 50 mM TEAB, 0.2% formic acid (FA), and then 50% acetonitrile (ACN)/0.2% FA, centrifuging the S-Trap plate at 1000× *g* for 2 min after each step. The peptides were dried down and resuspended in 40 µL of 2% ACN/0.1% trifluoroacetic acid (TFA). Peptide content was estimated using the Pierce Quantitative Colorimetric Peptide Assay (Thermo Scientific, Waltham, MA, USA) prior to nLC-MS/MS analysis.

#### 3.2.3. LC-MS/MS Analysis

The data were acquired using the data-dependent acquisition (DDA) mode in an UltiMate 3000 RSLCnano system coupled with the high-resolution Q Exactive HF-X mass spectrometer (Thermo Fisher Scientific, San José, CA, USA) to guarantee retention time reproducibility and mass accuracy. The full MS scan was set with an acquisition range of *m*/*z* 375–1500, a resolution of 120,000 (at *m*/*z* 200), a target AGC value of 3 × 10^6^, and a maximum injection time (IT) of 100 ms. The top 20 precursors were fragmented with a normalized collision energy (NCE) of 28. For the MS2 acquisition, the instrument was set with a resolution of 15,000 (at *m*/*z* 200), a target AGC value of 1 × 10^6^, a maximum IT of 50 ms, and an isolation window of 1.2 *m*/*z*. Dynamic exclusion was 40 s. Approximately 2 µg of peptides were analyzed for each sample with at least 2 replicates. Peptide elution was performed with a gradient of ACN and FA for 120 min, using the trap column C18 Acclaim PepMap^TM^ 100 (2 cm × 75 μm i.d.; 100 Å) and the column PepMap^TM^ RSLC C18 (2 µm, 100 Å, 75 µm i.d. × 50 cm).

#### 3.2.4. Data Analysis

Data analysis was performed on Proteome Discoverer 2.4 (Thermo Scientific, San José, CA, USA). For peptide identification, MSPepSearch was used against the Human spectral library ProteomeTools_HCD28_PD using a UniProt human database (Date: 28 January 2020). SEQUEST HT was also used against the same UniProt human database for unassigned peptides from MSPepSearch. For the peptide search, cysteine carbamidomethylation was set as a static modification, methionine oxidation as a dynamic modification, and acetylation, methionine loss (met-loss), and met-loss plus acetylation as a dynamic modification in the protein terminus. The precursor and fragment mass tolerance were set at 10 ppm and 0.02 ppm, respectively, and up to 2 missed cleavages were allowed. The confidence level used was FDR < 0.01 at the peptide level and FDR < 0.05 at the protein level. The node Feature Mapper was used in the consensus workflow for chromatographic alignment and identification of peptides based on MS1 information. For chromatographic alignment, the maximum RT shift was set at 3 min and the mass tolerance at 10 ppm. For feature linking and mapping, the RT and mass tolerance were set at 0 min and 0 ppm, respectively, the default for Proteome Discoverer, and the minimum S/N threshold was 5. Peptide and protein quantifications were performed based on the Label-free quantification approach using the precursor ion intensity to infer peptide abundance, and considering all peptides to calculate the abundance at the protein level.

#### 3.2.5. Bioinformatic Analysis

For data analysis, the proteins identified in at least 1 technical replicate were included. The characterization of undepleted and depleted plasma proteome was performed using the DAVID functional annotation tool, analyzing the functional category “UP_KEYWORDS” and “KEGG Pathway” and considering the results with a *p*-value and FDR < 0.05. The quantitative analysis was performed with the Perseus 1.6.12.0 software. The data were transformed by log2, normalized by subtracting the median, and filtered by 1 valid value in each group. The coefficient variation (CV) between experiments was determined considering the lognormal distribution of the MS experiment results [83,84,85]. The heatmap was built using the mean values and normalizing the proteins by Z-score. The construction of boxplot graphs was performed with the GraphPad Prism. 8.3.1 software using the estimated concentration of proteins in the blood available in the The Human Protein Atlas database for plasma proteins (available at https://www.proteinatlas.org/humanproteome/blood+protein, accessed on: 5 March 2020). The proteins were annotated using the information available in the same database (available at https://www.proteinatlas.org/search/protein_class:Plasma+proteins, accessed on: 29 July 2020) [45,46,47].

### 3.3. Evaluation of MS1-Transferring Efficiency-HeLa Digest Dilution Series

The peptide dilution series were prepared from commercial HeLa digest (Pierce^TM^ HeLa Protein Digest Standard, Thermo Scientific, Waltham, MA, USA). The analysis considered the following concentrations: 1000, 500, 200, 100, 40, 20, and 10 ng/μL. Each dilution was analyzed by LC-MS/MS by injecting 1 μL of solution. In the case of dilutions of DIA analysis, samples were spiked in with iRT Kit peptides (Biognosys, Schlieren, Switzerland) for retention time normalization. All analyses were performed in triplicate. Sample loading, separation, and data acquisition were performed in the same LC-MS/MS system described previously. Samples were separated by 97 min gradients whereby data were acquired by both DDA and DIA modes. In DDA, the instrument was set as follows: full MS in a range of *m*/*z* 375–1500, resolution of 120,000 (at *m*/*z* 200), target AGC value of 3 × 10^6^, and maximum injection time (IT) of 50 ms. The top 20 precursors were fragmented with an NCE of 28. MS2 acquisition was set with a resolution of 15,000 (at *m*/*z* 200), a target AGC value of 1 × 10^5^, maximum IT of 19 ms, and an isolation window of 1.2 *m*/*z*. Dynamic exclusion was set to 40 s. For DIA acquisition, the instrument was set as follows: full-MS scan parameters were kept the same as described for DDA experiments; for fragmentation analysis, the NCE was set at 28, resolution at 30,000 (at *m*/*z* 200), the AGC target value at 1 × 10^6^, and the MSX count and isolation window at 18 and *m*/*z* 16, respectively. Data from DDA were analyzed using the same parameters described previously. For DIA, protein identification was performed in Spectronaut (Biognosys, Schlieren, Switzerland) with the following parameters: chromatogram alignment and RT calibration were performed with the iRT Biognosys’ kit; the MS quantity level was set as MS1; the quantity type was set as the height and precursor; and protein q-value cutoff was 1%. The spectral library was built in Spectronaut (Biognosys, Schlieren, Switzerland) using all Proteome Discoverer results from the DDA data.

To demonstrate the linear nature of the MS1 transfers, a linear regression analysis per protein was performed, where the MS intensities (log2 expression values) were used as a response variable and the dilution points as an independent variable. The rate of false discoveries was analyzed by following a target–decoy strategy [86]. A decoy set of proteins was created by randomizing the MS intensities from the original set of proteins (target) so that each protein adopted an incorrect intensity value. The linear regression analysis was repeated using both sets of proteins (target + decoy) and the R2 parameter was used as a score to determine the FDR. In this case, an FDR threshold of 5% was set and these proteins were considered to be truly linearly transferred. Linear regression analyses were performed using the R software [87,88].

### 3.4. Assessment of Plasma Proteome of MM Patients Using WiMT

#### 3.4.1. Sample Description

A pool (*n* = 57) of MM samples from patients at different stages of the disease (as described in the The Human Melanoma Proteome Atlas [65,89]) and 10 individual samples from MM patients in the early stage (primary tumor) of disease were analyzed. All patients had undergone surgical resection of their tumors and subsequent histopathological characterization supported by imaging studies. Table 2 displays the clinicopathological properties of the herein analyzed patients.

#### 3.4.2. Sample Digestion

EDTA plasma samples were diluted with MilliQ water (1:10), and an aliquot of 8.75 µL (~70 µg of protein) of each sample was separated using the S-trap protein digestion protocol. For sample reduction, 42.25 µL of 10% SDS/25 mM DTT in 100 mM TEAB solution was added to the diluted plasma. The reduction, alkylation, and digestion steps were performed as described previously [82].

#### 3.4.3. LC-MS/MS Analysis

The samples were loaded, separated, and analyzed in the same system, as described for custom database development. The elution gradient and the parameters for data acquisition were kept the same.

#### 3.4.4. Data Analysis

The data analysis was performed using the same parameters described previously. The proteomap analysis was performed using the online tool Bionic Visualizations-Proteomaps (*Homo sapiens* database) [90,91,92]. For MS1t, all depleted samples were processed together with the undepleted ones in the same workflow. To determine the FDR of the MS1 transfer, a target–decoy strategy was followed [86]. A decoy set of proteins was created by simulating a distribution of values similar to that followed by the MS1 intensities of the transferred proteins (target dataset). To calculate the FDR values, an empirical score [20,93,94] per protein was created based on the protein intensity (Ii), the protein abundance rank (proteins sorted by ascending MS intensities, Rank1), and the probability of being a plasma protein. The probability of being a plasma protein (PDi) was determined based on cumulative distribution functions that utilized the previous identification of plasma proteins taken from both public repositories (Table S8) and in-house experiments (Table S9). Finally, the empirical scoring scheme was applied (Figure S6) for both the decoy and target proteins datasets with an FDR threshold set at 5%. Proteins with FDR < 0.05 were considered correctly transferred. The analysis was performed using R software [87,88] and SPSS Statistics 21.0 (IBM, Somers, IL, USA). Bioinformatic analysis was performed as previously described for the characterization of the custom database.

## 4. Conclusions

We established the MPP of undepleted patient samples using our newly developed WiMT strategy, mapping more than 1200 proteins and 10,000 peptides in non-depleted plasma samples from MM patients. The MPP is mainly characterized by proteins related to cancer pathway signaling processes, the immune system, genetic information processing (protein folding, sorting, and degradation), cellular processes (protein transport) and the biosynthesis of metabolites. These results represent the proteins and processes that could be followed by the proteomic analysis of undepleted plasma from melanoma patients. Our results show great potential for large-scale screening in melanoma proteomics studies, providing an invaluable tool for monitoring blood proteins in melanoma patients. The developments are generic and can be applied to other neoplastic diseases.

## Figures and Tables

**Table 1:** Potential plasma/serum malignant melanoma biomarkers.

Description	FDA Biomarkers	Identified in the Pools with WiMT	Custom Database				% MM Patients	Reference
			Pool	Low-Dep	Mid-Dep	Deep-Dep		
Lactate dehydrogenase	x	x	x	x	x	x	100	[73]
Tyrosinase								[73]
Vascular endothelial growth factor								[73]
Osteopontin		x				x	40	[73]
YKL-40, Chitinase-3-like protein 1		x			x	x	100	[73]
Melanoma-inhibitory activityprotein								[73]
S100B								[73]
Interleukin-8								[73]
CD44 antigen		x	x	x	x	x	100	[74]
Laminin		x			x	x	100	[74]
Tenascin C		x				x		[74]
Collagen type VI		x		x	x	x	100	[74]
Melanoma cell adhesion molecule (MCAM)		x	x	x	x	x	100	[75]
Galectin-3 binding protein		x	x	x	x	x	100	[76]
Endostatin- Collagen alpha-1 (XVIII) chain		x	x	x	x	x	100	[76]
C-reactive protein	x	x	x	x	x	x	100	[77]
Serum amyloid A		x	x	x	x	x	100	[7]

List of proteins considered as MM biomarkers candidates in plasma or serum by previous works. Among the 17 proteins, lactate dehydrogenase and C-reactive protein have been approved by the FDA. Using the WiMT strategy, we were able to identify 12 proteins in the pools and 11 in individual plasma from MM patients. The immunodepleted strategies were important for the identification of osteopontin, chitinase-3-like protein 1, laminin, tenascin C, and collagen type VI. The percentage of MM patients that had proteins identified with WiMT was calculated, and most were identified in all the 10 patients.

**Table 2:** Clinicopathological data of the 10 MM patients. Primary tumor samples from the 10 MM patients were submitted for histopathological characterization after tumor resection. T, N, M system classification: T (Primary tumor), N (Regional Lymph Nodes, M (Distant Metastasis).

Patients	Patient Code	Age	Gender	Breslow	Clark Level	Type of Tumor	Main Cell Type	T	N	M	Stage	Type of Treatment	Systemic Treatment
Patient 1	PTP054	70	Female	2.248	IV	SSM	Naevoid	3b	0	0	IIB	Adjuvant	Interferon alfa
Patient 2	PTP048	68	Female	13.19	IV	SSM	Naevoid	4b	0	0	IIC	Adjuvant	Interferon alfa
Patient 3	PTP050	73	Male	7.36	IV	Unclassified	Naevoid	4b	1b	0	IIIB	None	None
Patient 4	PTP068	75	Female	65	IV	NM	NaevoidSpindle	4a	0	0	IIC	Adjuvant	Interferon alfa
Patient 5	PTP007	74	Female	8.14	IV	Unclassified	Naevoid	4a	0	0	IIB	None	None
Patient 6	PTP027	69	Male	4.36	V	ALM	Spindle	4b	0	0	IIC	None	None
Patient 7 *	PTP044	80	Male	9.86	IV	SSM	Spindle	4a		0		None	None
Patient 8	PTP039	84	Male	3.208	IV	ALM	Naevoid	3a	0	0	IIA	None	None
Patient 9	PTP028	25	Male	11.84	IV	NM	Naevoid	4b	0	0	IIC	None	None
Patient 10	PTP029	83	Female	0.386	II	SSM	Naevoid	1a	0	0	IA	None	None

* The full classification of patients 7 was not possible as the examination was not fully completed.

## Data Availability

The data files (ProteomeXchange Id: PXD028709) have been deposited at PRIDE—the Proteomics Identification Database.

## Supplementary Materials

The following are available online at https://www.mdpi.com/article/10.3390/cancers13246224/s1. Figure S1: Linearity analysis of protein abundance groups depending on the protein amount analyzed. Figure S2: Comparison of the results obtained by MS1t, DDA, and DIA according to the range of protein abundance. Figure S3: Correlation analysis between pool replicates and protein blood concentration. Figure S4: Pearson correlation analysis among the results from the 10 MM patients. Figure S5A–C: Chromatograms obtained from the immunedepletion strategies. Figure S6: Empirical scores used for MS1 transfer evaluation. Table S1: Custom database description. Classification of proteins based on the depletion levels. Table S2: Cluster B proteins depleted by the SuperMix strategy. Table S3: Summary of functional annotation analysis results for the undepleted plasma and the 3 immune depletion approaches. Table S4: KEGG pathway analysis results for the undepleted plasma and the 3 immune depletion approaches. Table S5: Peptide identification in the pool of plasma samples from MM patients. Table S6: Peptide identification in plasma samples from the 10 MM patients. Table S7: Proteins identified in the analysis of MM pool and primary tumor patients by MS2 and WiMT. Table S8: Probability of proteins from public repository being plasma proteins. Table S9: Probability of proteins from in-house experiments being plasma proteins.

## References

[1] Davis L.E., Shalin S.C., Tackett A.J. (2019). Current state of melanoma diagnosis and treatment. Cancer Biol. Ther..

[2] Geyer P.E., Holdt L.M., Teupser D., Mann M. (2017). Revisiting biomarker discovery by plasma proteomics. Mol. Syst. Biol..

[3] Zhang H., Liu A.Y., Loriaux P., Wollscheid B., Zhou Y., Watts J.D., Aebersold R. (2007). Mass Spectrometric Detection of Tissue Proteins in Plasma. Mol. Cell. Proteom..

[4] Anderson N.L. (2002). The Human Plasma Proteome: History, Character, and Diagnostic Prospects. Mol. Cell. Proteom..

[5] Nanjappa V., Thomas J.K., Marimuthu A., Muthusamy B., Radhakrishnan A., Sharma R., Khan A.A., Balakrishnan L., Sahasrabuddhe N.A., Kumar S. (2014). Plasma Proteome Database as a resource for proteomics research: 2014 update. Nucleic Acids Res..

[6] Tirumalai R.S., Chan K.C., Prieto D.A., Issaq H.J., Conrads T.P., Veenstra T.D. (2003). Characterization of the Low Molecular Weight Human Serum Proteome. Mol. Cell. Proteom..

[7] Findeisen P., Zapatka M., Peccerella T., Matzk H., Neumaier M., Schadendorf D., Ugurel S. (2009). Serum Amyloid A As a Prognostic Marker in Melanoma Identified by Proteomic Profiling. J. Clin. Oncol..

[8] Mian S., Ugurel S., Parkinson E., Schlenzka I., Dryden I., Lancashire L., Ball G., Creaser C., Rees R., Schadendorf D. (2005). Serum Proteomic Fingerprinting Discriminates Between Clinical Stages and Predicts Disease Progression in Melanoma Patients. J. Clin. Oncol..

[9] Greco M., de Mitri M., Chiriacò F., Leo G., Brienza E., Maffia M. (2009). Serum proteomic profile of cutaneous malignant melanoma and relation to cancer progression: Association to tumor derived alpha-N-acetylgalactosaminidase activity. Cancer Lett..

[10] Lee P.Y., Osman J., Low T.Y., Jamal R. (2019). Plasma/serum proteomics: Depletion strategies for reducing high-abundance proteins for biomarker discovery. Bioanalysis.

[11] Muqaku B., Eisinger M., Meier S.M., Tahir A., Pukrop T., Haferkamp S., Slany A., Reichle A., Gerner C. (2017). Multi-omics Analysis of Serum Samples Demonstrates Reprogramming of Organ Functions Via Systemic Calcium Mobilization and Platelet Activation in Metastatic Melanoma. Mol. Cell. Proteom..

[12] Babačić H., Lehtiö J., de Coaña Y.P., Pernemalm M., Eriksson H. (2020). In-depth plasma proteomics reveals increase in circulating PD-1 during anti-PD-1 immunotherapy in patients with metastatic cutaneous melanoma. J. Immunother. Cancer.

[13] Keshishian H., Burgess M.W., Gillette M.A., Mertins P., Clauser K., Mani D.R., Kuhn E.W., Farrell L.A., Gerszten R.E., Carr S.A. (2015). Multiplexed, Quantitative Workflow for Sensitive Biomarker Discovery in Plasma Yields Novel Candidates for Early Myocardial Injury. Mol. Cell. Proteom..

[14] Pernemalm M., Sandberg A., Zhu Y., Boekel J., Tamburro D., Schwenk J.M., Björk A., Wahren-Herlenius M., Åmark H., Östenson C.-G. (2019). In-depth human plasma proteome analysis captures tissue proteins and transfer of protein variants across the placenta. eLife.

[15] Park J., Kim H., Kim S.Y., Kim Y., Lee J.-S., Dan K., Seong M.-W., Han D. (2020). In-depth blood proteome profiling analysis revealed distinct functional characteristics of plasma proteins between severe and non-severe COVID-19 patients. Sci. Rep..

[16] Smith M.P.W., Wood S.L., Zougman A., Ho J.T.C., Peng J., Jackson D., Cairns D.A., Lewington A.J.P., Selby P.J., Banks R.E. (2011). A systematic analysis of the effects of increasing degrees of serum immunodepletion in terms of depth of coverage and other key aspects in top-down and bottom-up proteomic analyses. Proteomics.

[17] Macklin A., Khan S., Kislinger T. (2020). Recent advances in mass spectrometry based clinical proteomics: Applications to cancer research. Clin. Proteom..

[18] Geyer P.E., Kulak N.A., Pichler G., Holdt L.M., Teupser D., Mann M. (2016). Plasma Proteome Profiling to Assess Human Health and Disease. Cell Syst..

[19] Geyer P.E., Voytik E., Treit P.V., Doll S., Kleinhempel A., Niu L., Müller J.B., Buchholtz M., Bader J.M., Teupser D. (2019). Plasma Proteome Profiling to detect and avoid sample-related biases in biomarker studies. EMBO Mol. Med..

[20] Perez-Riverol Y., Sánchez A., Noda J., Borges D., Carvalho P.C., Wang R., Vizcaíno J.A., Betancourt L., Ramos Y., Duarte G. (2013). HI-Bone: A Scoring System for Identifying Phenylisothiocyanate-Derivatized Peptides Based on Precursor Mass and High Intensity Fragment Ions. Anal. Chem..

[21] Borges D., Perez-Riverol Y., Nogueira F.C.S., Domont G.B., Noda J., Leprevost F.D.V., Besada V., França F.M.G., Barbosa V.C., Sánchez A. (2013). Effectively addressing complex proteomic search spaces with peptide spectrum matching. Bioinformatics.

[22] Betancourt L.H., Sánchez A., Pérez Y., de Cossío J.F., Gil J., Toledo P., Iguchi S., Aimoto S., González L.J., Padrón G. (2011). Charge state-selective separation of peptides by reversible modification of amino groups and strong cation-exchange chromatography: Evaluation in proteomic studies using peptide-centric database searches. J. Proteom..

[23] Sánchez A., Perez-Riverol Y., González L.J., Noda J., Betancourt L., Ramos Y., Gil J., Vera R., Padrón G., Besada V. (2010). Evaluation of Phenylthiocarbamoyl-Derivatized Peptides by Electrospray Ionization Mass Spectrometry: Selective Isolation and Analysis of Modified Multiply Charged Peptides for Liquid Chromatography−Tandem Mass Spectrometry Experiments. Anal. Chem..

[24] Yang Y., Liu X., Shen C., Lin Y., Yang P., Qiao L. (2020). In silico spectral libraries by deep learning facilitate data-independent acquisition proteomics. Nat. Commun..

[25] Chapman J.D., Goodlett D.R., Masselon C.D. (2014). Multiplexed and data-independent tandem mass spectrometry for global proteome profiling. Mass Spectrom. Rev..

[26] Richards A.L., Merrill A., Coon J.J. (2015). Proteome sequencing goes deep. Curr. Opin. Chem. Biol..

[27] Meier F., Geyer P.E., Winter S.V., Cox J., Mann M. (2018). BoxCar acquisition method enables single-shot proteomics at a depth of 10,000 proteins in 100 minutes. Nat. Methods.

[28] May D., Fitzgibbon M., Liu Y., Holzman T., Eng J., Kemp C.J., Whiteaker J., Paulovich A., McIntosh M. (2007). A Platform for Accurate Mass and Time Analyses of Mass Spectrometry Data. J. Proteome Res..

[29] Zimmer J.S., Monroe M.E., Qian W.-J., Smith R. (2006). Advances in proteomics data analysis and display using an accurate mass and time tag approach. Mass Spectrom. Rev..

[30] Moruz L., Hoopmann M.R., Rosenlund M., Granholm V., Moritz R.L., Käll L. (2013). Mass Fingerprinting of Complex Mixtures: Protein Inference from High-Resolution Peptide Masses and Predicted Retention Times. J. Proteome Res..

[31] Pasa-Tolić L., Masselon C., Barry R.C., Shen Y., Smith R.D. (2004). Proteomic analyses using an accurate mass and time tag strategy. BioTechniques.

[32] Cox J., Hein M., Luber C.A., Paron I., Nagaraj N., Mann M. (2014). Accurate Proteome-wide Label-free Quantification by Delayed Normalization and Maximal Peptide Ratio Extraction, Termed MaxLFQ. Mol. Cell. Proteom..

[33] Lim M.Y., Paulo J.A., Gygi S.P. (2019). Evaluating False Transfer Rates from the Match-between-Runs Algorithm with a Two-Proteome Model. J. Proteome Res..

[34] Byrling J., Kristl T., Hu D., Pla I., Sanchez A., Sasor A., Andersson R., Marko-Varga G., Andersson B. (2020). Mass spectrometry-based analysis of formalin-fixed, paraffin-embedded distal cholangiocarcinoma identifies stromal thrombospondin-2 as a potential prognostic marker. J. Transl. Med..

[35] Virant-Klun I., Leicht S., Hughes C., Krijgsveld J. (2016). Identification of Maturation-Specific Proteins by Single-Cell Proteomics of Human Oocytes. Mol. Cell. Proteom..

[36] Zhang B., Käll L., Zubarev R.A. (2016). DeMix-Q: Quantification-Centered Data Processing Workflow. Mol. Cell. Proteom..

[37] Röst H., Sachsenberg T., Aiche S., Bielow C., Weisser H., Aicheler F., Andreotti S., Ehrlich H.-C., Gutenbrunner P., Kenar E. (2016). OpenMS: A flexible open-source software platform for mass spectrometry data analysis. Nat. Methods.

[38] Kelemen O., Pla I., Sanchez A., Rezeli M., Szasz A.M., Malm J., Laszlo V., Kwon H.J., Dome B., Marko-Varga G. (2020). Proteomic analysis enables distinction of early-versus advanced-stage lung adenocarcinomas. Clin. Transl. Med..

[39] Poulsen L.L.C., Pla I., Sanchez A., Grøndahl M.L., Marko-Varga G., Andersen C.Y., Englund A.L.M., Malm J. (2019). Progressive changes in human follicular fluid composition over the course of ovulation: Quantitative proteomic analyses. Mol. Cell. Endocrinol..

[40] Tu C., Rudnick P.A., Martinez M.Y., Cheek K.L., Stein S.E., Slebos R.J.C., Liebler D.C. (2010). Depletion of Abundant Plasma Proteins and Limitations of Plasma Proteomics. J. Proteome Res..

[41] Gong Y., Li X., Yang B., Ying W., Li D., Zhang Y., Dai S., Cai Y., Wang J., He A.F. (2006). Different Immunoaffinity Fractionation Strategies to Characterize the Human Plasma Proteome. J. Proteome Res..

[42] Roche S., Tiers L., Provansal M., Séveno M., Piva M.-T., Jouin P., Lehmann S. (2009). Depletion of one, six, twelve or twenty major blood proteins before proteomic analysis: The more the better?. J. Proteom..

[43] Shi T., Zhou J.-Y., Gritsenko M.A., Hossain M., Camp D.G., Smith R.D., Qian W.-J. (2012). IgY14 and SuperMix immunoaffinity separations coupled with liquid chromatography–mass spectrometry for human plasma proteomics biomarker discovery. Methods.

[44] Qian W.-J., Kaleta D.T., Petritis B.O., Jiang H., Liu T., Zhang X., Mottaz H.M., Varnum S.M., Camp D.G., Huang L. (2008). Enhanced Detection of Low Abundance Human Plasma Proteins Using a Tandem IgY12-SuperMix Immunoaffinity Separation Strategy. Mol. Cell. Proteom..

[45] Uhlén M., Karlsson M.J., Hober A., Svensson A.-S., Scheffel J., Kotol D., Zhong W., Tebani A., Strandberg L., Edfors F. (2019). The human secretome. Sci. Signal..

[46] Uhlen M., Oksvold P., Fagerberg L., Lundberg E., Jonasson K., Forsberg M., Zwahlen M., Kampf C., Wester K., Hober S. (2010). Towards a knowledge-based Human Protein Atlas. Nat. Biotechnol..

[47] Pontén F., Jirström K., Uhlen M. (2008). The Human Protein Atlas—A tool for pathology. J. Pathol..

[48] Liu Z., Fan S.-H., Liu H., Yu J., Qiao R., Zhou M., Yang Y., Zhou J., Xie P. (2016). Enhanced Detection of Low-Abundance Human Plasma Proteins by Integrating Polyethylene Glycol Fractionation and Immunoaffinity Depletion. PLoS ONE.

[49] Khan A. (2012). Detection and quantitation of forty eight cytokines, chemokines, growth factors and nine acute phase proteins in healthy human plasma, saliva and urine. J. Proteom..

[50] Tandler N., Mosch B., Pietzsch J. (2012). Protein and non-protein biomarkers in melanoma: A critical update. Amino Acids.

[51] Bansal A., Celeste Simon M. (2018). Glutathione metabolism in cancer progression and treatment resistance. J. Cell Biol..

[52] Smeyne M., Smeyne R.J. (2013). Glutathione metabolism and Parkinson’s disease. Free. Radic. Biol. Med..

[53] Nugent A.A., Kolpak A.L., Engle E.C. (2012). Human disorders of axon guidance. Curr. Opin. Neurobiol..

[54] Vettore L., Westbrook R., Tennant D.A. (2020). New aspects of amino acid metabolism in cancer. Br. J. Cancer.

[55] Lukey M.J., Katt W.P., Cerione R.A. (2017). Targeting amino acid metabolism for cancer therapy. Drug Discov. Today.

[56] Ruocco M.R., Avagliano A., Granato G., Vigliar E., Masone S., Montagnani S., Arcucci A. (2019). Metabolic flexibility in melanoma: A potential therapeutic target. Semin. Cancer Biol..

[57] Wu Z., Wu J., Zhao Q., Fu S., Jin J. (2020). Emerging roles of aerobic glycolysis in breast cancer. Clin. Transl. Oncol..

[58] Davies M.A. (2012). The Role of the PI3K-AKT Pathway in Melanoma. Cancer J..

[59] Li L., Posch A. (2015). Dynamic Range Compression with ProteoMiner™: Principles and Examples. Proteomic Profiling. Methods in Molecular Biology.

[60] Bekker-Jensen D.B., Kelstrup C.D., Batth T.S., Larsen S.C., Haldrup C., Bramsen J.B., Sørensen K.D., Høyer S., Ørntoft T.F., Andersen C.L. (2017). An Optimized Shotgun Strategy for the Rapid Generation of Comprehensive Human Proteomes. Cell Syst..

[61] Bian Y., Zheng R., Bayer F.P., Wong C., Chang Y.-C., Meng C., Zolg D.P., Reinecke M., Zecha J., Wiechmann S. (2020). Robust, reproducible and quantitative analysis of thousands of proteomes by micro-flow LC–MS/MS. Nat. Commun..

[62] Cong Y., Liang Y., Motamedchaboki K., Huguet R., Truong T., Zhao R., Shen Y., Lopez-Ferrer D., Zhu Y., Kelly R.T. (2020). Improved Single-Cell Proteome Coverage Using Narrow-Bore Packed NanoLC Columns and Ultrasensitive Mass Spectrometry. Anal. Chem..

[63] Ivanov M.V., Bubis J.A., Gorshkov V., Abdrakhimov D.A., Kjeldsen F., Gorshkov M.V. (2021). Boosting MS1-only Proteomics with Machine Learning Allows 2000 Protein Identifications in Single-Shot Human Proteome Analysis Using 5 min HPLC Gradient. J. Proteome Res..

[64] Geiger T., Wehner A., Schaab C., Cox J., Mann M. (2012). Comparative Proteomic Analysis of Eleven Common Cell Lines Reveals Ubiquitous but Varying Expression of Most Proteins. Mol. Cell. Proteom..

[65] Betancourt L.H., Gil J., Sanchez A., Doma V., Kuras M., Murillo J.R., Velasquez E., Çakır U., Kim Y., Sugihara Y. (2021). The Human Melanoma Proteome Atlas—Complementing the melanoma transcriptome. Clin. Transl. Med..

[66] Kato G.J., McGowan V., Machado R., Little J.A., Taylor J., Morris C.R., Nichols J.S., Wang X., Poljakovic M., Morris J.S.M. (2006). Lactate dehydrogenase as a biomarker of hemolysis-associated nitric oxide resistance, priapism, leg ulceration, pulmonary hypertension, and death in patients with sickle cell disease. Blood.

[67] Armstrong A.J., George D.J., Halabi S. (2010). Serum lactate dehydrogenase (LDH) as a biomarker for survival with mTOR inhibition in patients with metastatic renal cell carcinoma (RCC). J. Clin. Oncol..

[68] Vuong N.L., Le Duyen H.T., Lam P.K., Tam D.T.H., Chau N.V.V., van Kinh N., Chanpheaktra N., Lum L.C.S., Pleités E., Jones N. (2020). C-reactive protein as a potential biomarker for disease progression in dengue: A multi-country observational study. BMC Med..

[69] Fond G., Lançon C., Auquier P., Boyer L. (2018). C-Reactive Protein as a Peripheral Biomarker in Schizophrenia. An Updated Systematic Review. Front. Psychiatry.

[70] Li H., Xiang X., Ren H., Xu L., Zhao L., Chen X., Long H., Wang Q., Wu Q. (2020). Serum Amyloid A is a biomarker of severe Coronavirus Disease and poor prognosis. J. Infect..

[71] Li Z., Hou Y., Zhao M., Li T., Liu Y., Chang J., Ren L. (2020). Serum amyloid a, a potential biomarker both in serum and tissue, correlates with ovarian cancer progression. J. Ovarian Res..

[72] Hara A., Niwa M., Noguchi K., Kanayama T., Niwa A., Matsuo M., Hatano Y., Tomita H. (2020). Galectin-3 as a Next-Generation Biomarker for Detecting Early Stage of Various Diseases. Biomolecules.

[73] Karagiannis P., Fittall M., Karagiannis S.N. (2014). Evaluating biomarkers in melanoma. Front. Oncol..

[74] Malaguarnera M. (2010). Serum markers of cutaneous melanoma. Front. Biosci..

[75] Eisenstein A., Gonzalez E.C., Raghunathan R., Xu X., Wu M., McLean E.O., McGee J., Ryu B., Alani R.M. (2018). Emerging Biomarkers in Cutaneous Melanoma. Mol. Diagn. Ther..

[76] Nyakas M., Aamdal E., Jacobsen K.D., Guren T.K., Aamdal S., Hagene K.T., Brunsvig P., Yndestad A., Halvorsen B., Tasken K.A. (2019). Prognostic biomarkers for immunotherapy with ipilimumab in metastatic melanoma. Clin. Exp. Immunol..

[77] Fang S., Wang Y., Sui D., Liu H., Ross M.I., Gershenwald J.E., Cormier J.N., Royal R.E., Lucci A., Schacherer C.W. (2015). C-Reactive Protein As a Marker of Melanoma Progression. J. Clin. Oncol..

[78] Yu F., Haynes S.E., Nesvizhskii A.I. (2021). IonQuant Enables Accurate and Sensitive Label-Free Quantification with FDR-Controlled Match-Between-Runs. Mol. Cell. Proteom..

[79] Perez-Riverol Y., Sánchez A., Ramos Y., Schmidt A., Müller M., Betancourt L., González L.J., Vera R., Padron G., Besada V. (2011). In silico analysis of accurate proteomics, complemented by selective isolation of peptides. J. Proteom..

[80] Malm J., Végvári Á., Rezeli M., Upton P., Danmyr P., Nilsson R., Steinfelder E., Marko-Varga G. (2012). Large scale biobanking of blood—The importance of high density sample processing procedures. J. Proteom..

[81] Malm J., Lindberg H., Erlinge D., Appelqvist R., Yakovleva M., Welinder C., Steinfelder E., Fehniger T.E., Marko-Varga G. (2015). Semi-automated biobank sample processing with a 384 high density sample tube robot used in cancer and cardiovascular studies. Clin. Transl. Med..

[82] Kuras M., Woldmar N., Kim Y., Hefner M., Malm J., Moldvay J., Döme B., Fillinger J., Pizzatti L., Gil J. (2021). Proteomic Workflows for High-Quality Quantitative Proteome and Post-Translational Modification Analysis of Clinically Relevant Samples from Formalin-Fixed Paraffin-Embedded Archives. J. Proteome Res..

[83] Canchola J.A., Tang S., Hemyari P., Paxinos E., Marins E. (2017). Correct Use of Percent Coefficient of Variation (%CV) Formula for Log-Transformed Data. MOJ Proteom. Bioinform..

[84] Koopmans L.H., Owen D.B., Rosenblatt J.I. (1964). Confidence intervals for the coefficient of variation for the normal and log normal distributions. Biometrika.

[85] Limpert E., Stahel W.A., Abbt M. (2001). Log-normal Distributions across the Sciences: Keys and Clues: On the charms of statistics, and how mechanical models resembling gambling machines offer a link to a handy way to characterize log-normal distributions, which can provide deeper insight into variability and probability—Normal or log-normal: That is the question. BioScience.

[86] Elias J.E., Gygi S.P. (2010). Target-Decoy Search Strategy for Mass Spectrometry-Based Proteomics. Proteome Bioinform..

[87] RStudio Team (2016). RStudio: Integrated Development for R.

[88] Team R Core (2016). R: A Language and Environment for Statistical Computing.

[89] Betancourt L.H., Gil J., Kim Y., Doma V., Çakır U., Sanchez A., Murillo J.R., Kuras M., Parada I.P., Sugihara Y. (2021). The human melanoma proteome atlas—Defining the molecular pathology. Clin. Transl. Med..

[90] Bernhardt J., Funke S., Hecker M.S.J. Visualizing Gene Expression Data via Voronoi Treemaps. Proceedings of the Sixth International Symposium on Voronoi Diagrams.

[91] Liebermeister W., Noor E., Flamholz A., Davidi D., Bernhardt J., Milo R. (2014). Visual account of protein investment in cellular functions. Proc. Natl. Acad. Sci. USA.

[92] Otto A., Bernhardt J., Meyer H., Schaffer M., Herbst F.-A., Siebourg J., Mäder U., Lalk M., Hecker M., Becher D. (2010). Systems-wide temporal proteomic profiling in glucose-starved Bacillus subtilis. Nat. Commun..

[93] Chalkley R.J. (2013). Improving Peptide Identification Using Empirical Scoring Systems. Mass Spectrometry Data Analysis in Pro-teomicols, Methods in Molecular Biology (Methods and Protocols).

[94] Ivanov M., Levitsky L., Lobas A., Panic T., Laskay Ü.A., Mitulović G., Schmid R., Pridatchenko M.L., Tsybin Y., Gorshkov M.V. (2014). Empirical Multidimensional Space for Scoring Peptide Spectrum Matches in Shotgun Proteomics. J. Proteome Res..

