# Supplementary material for: Mapping the Melanoma Plasma Proteome (MPP) Using Single-Shot Proteomics Interfaced with the WiMT Database
Source: Cancers (Basel). 2021 Dec 10;13(24):6224. doi: 10.3390/cancers13246224 (PMC8699267; doi:10.3390/cancers13246224)
Supplement: Supplementary file 1 [file cancers-13-06224-s001.zip › Supplementary figures Final.pdf]

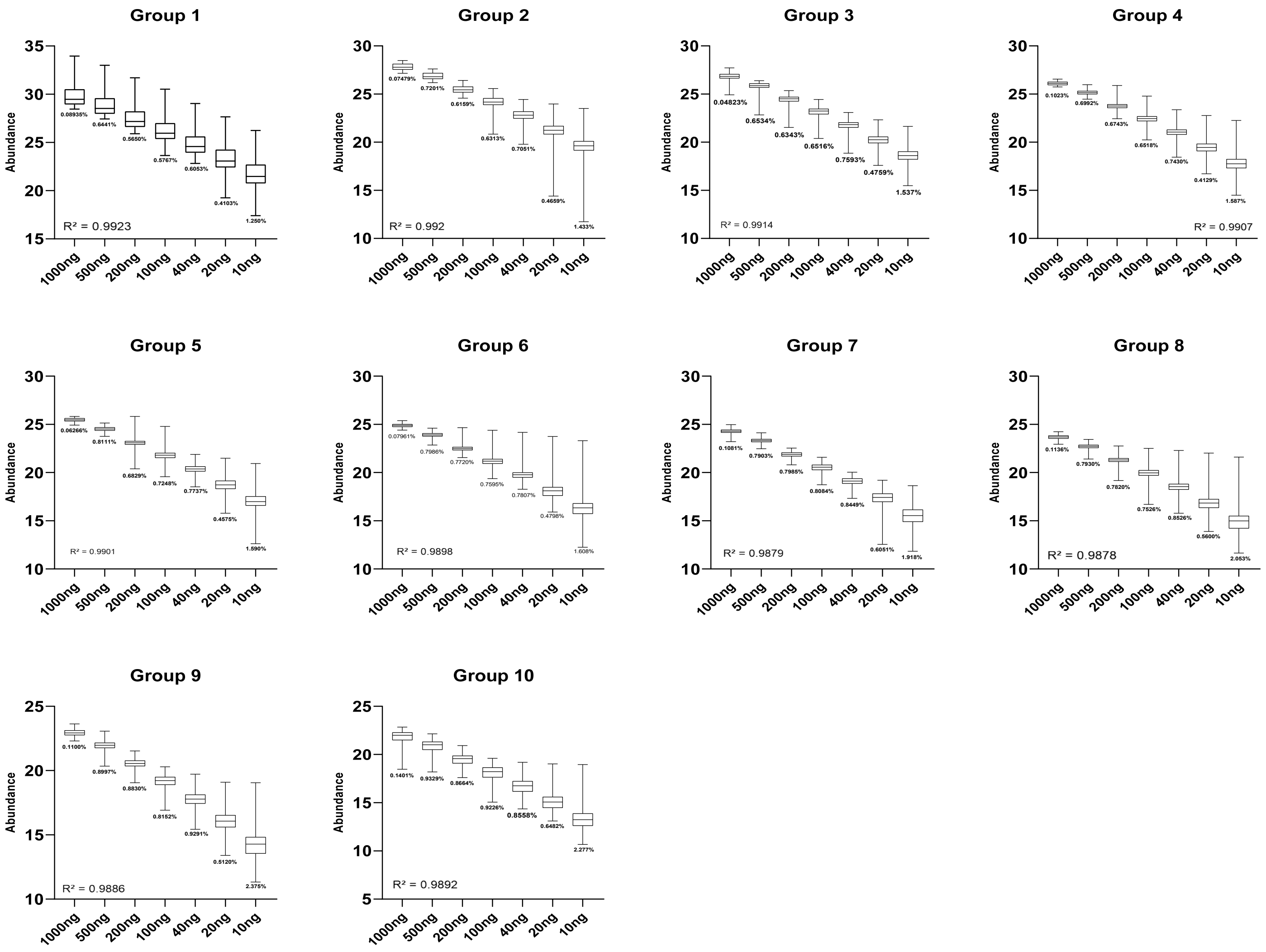

Figure S1: Linearity analysis of protein abundance groups depending on the protein amount analyzed. Abundance: Log2 (intensity). The number under the box plots represents the coefficient variation of the abundance medians in each experiment.

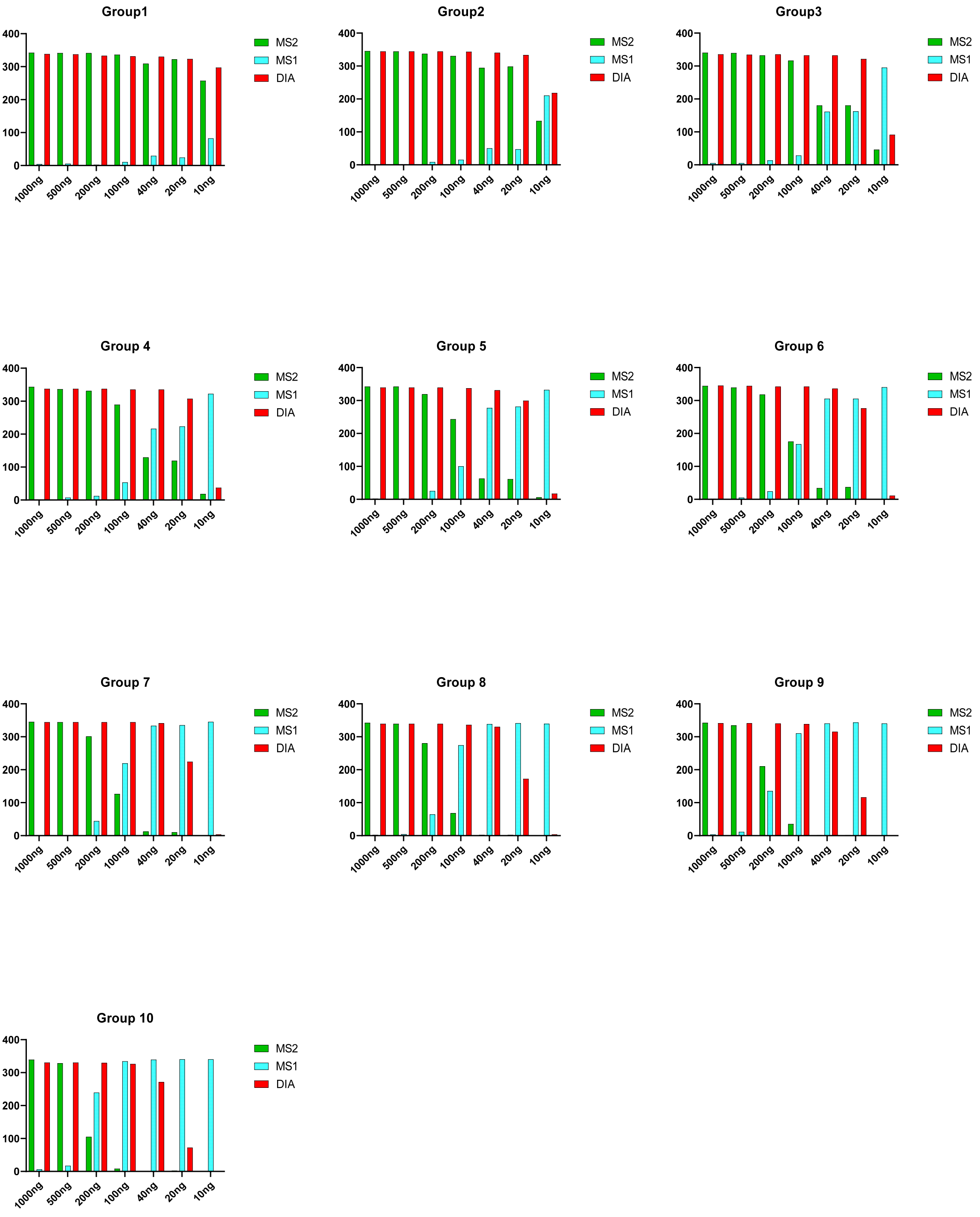

Figure S2: Comparison of the results obtained by MS1t, DDA and DIA according to the range of protein abundance. The proteins were ranked by abundance and divided in ten groups. Group 1 represents the most abundant proteins and Group 10 the less abundant proteins.

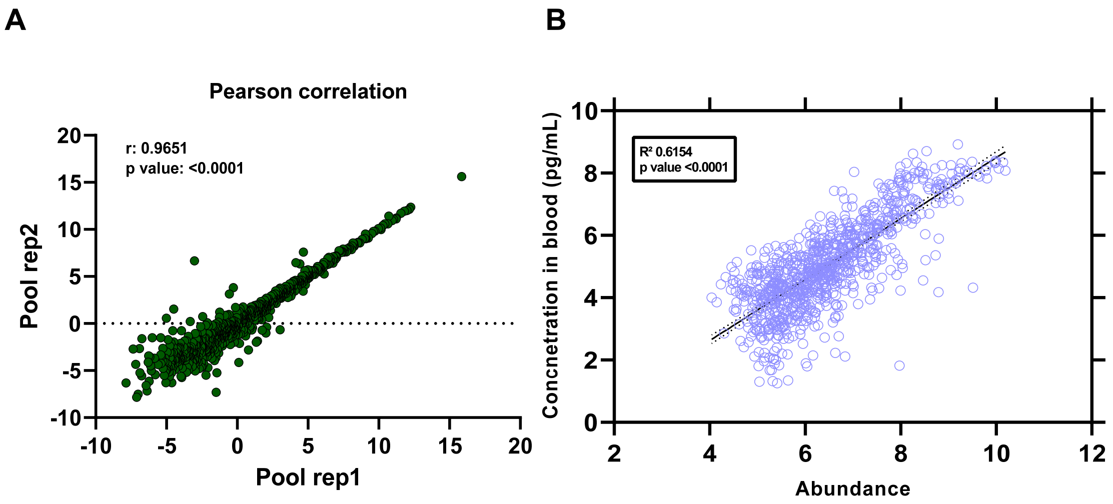

Figure S3: Correlation analysis between pool replicates and protein blood concentration. (A) Pearson correlation between the two technical replicates for the analysis of the pool of samples from MM patients using MS1t. (B) Correlation between protein intensity and its respective estimated concentration in blood available in ´The Human Protein Atlas´ database.

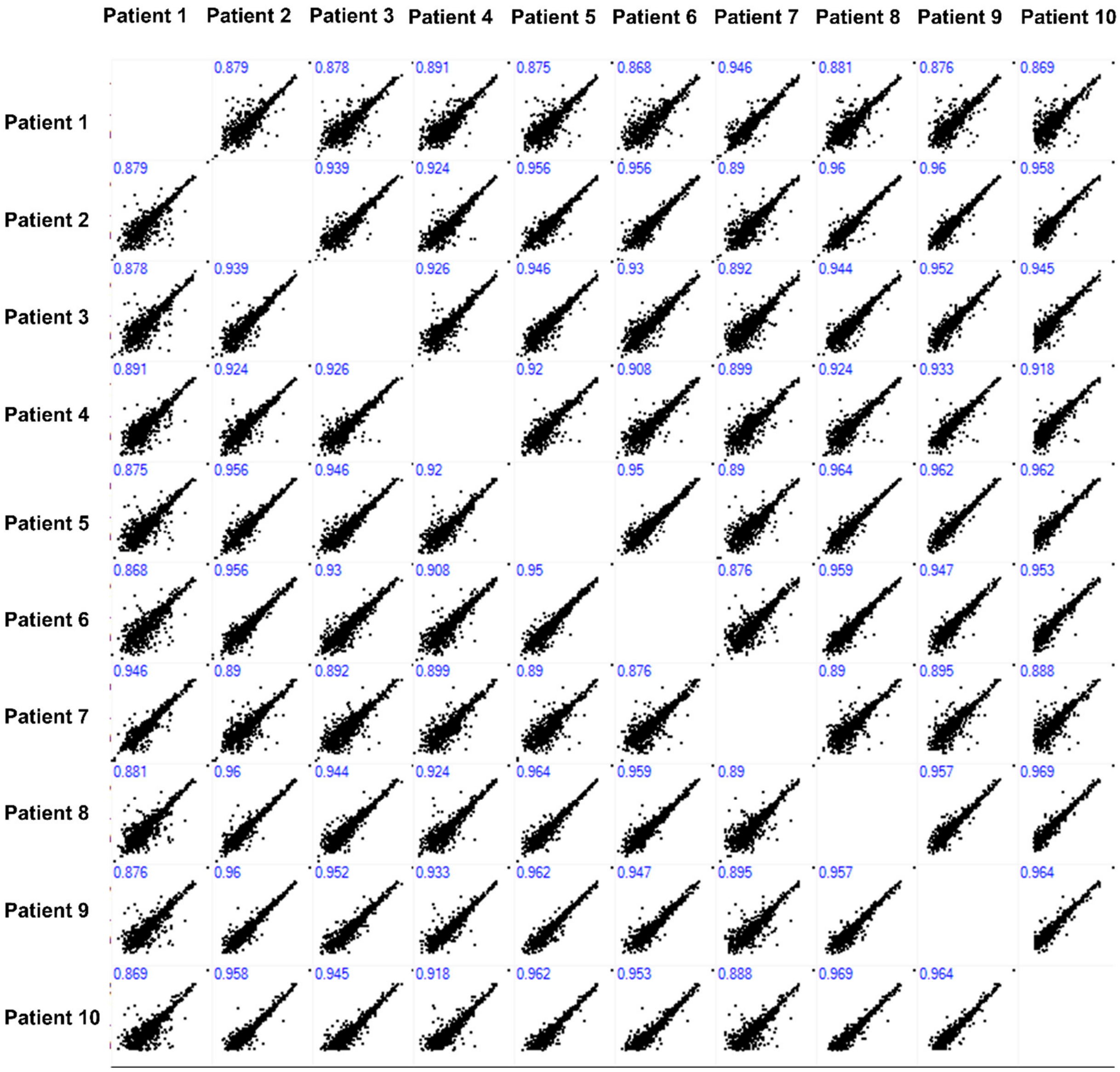

Figure S4: Pearson correlation analysis among the results from the 10 MM patients. The r value is represented in blue.

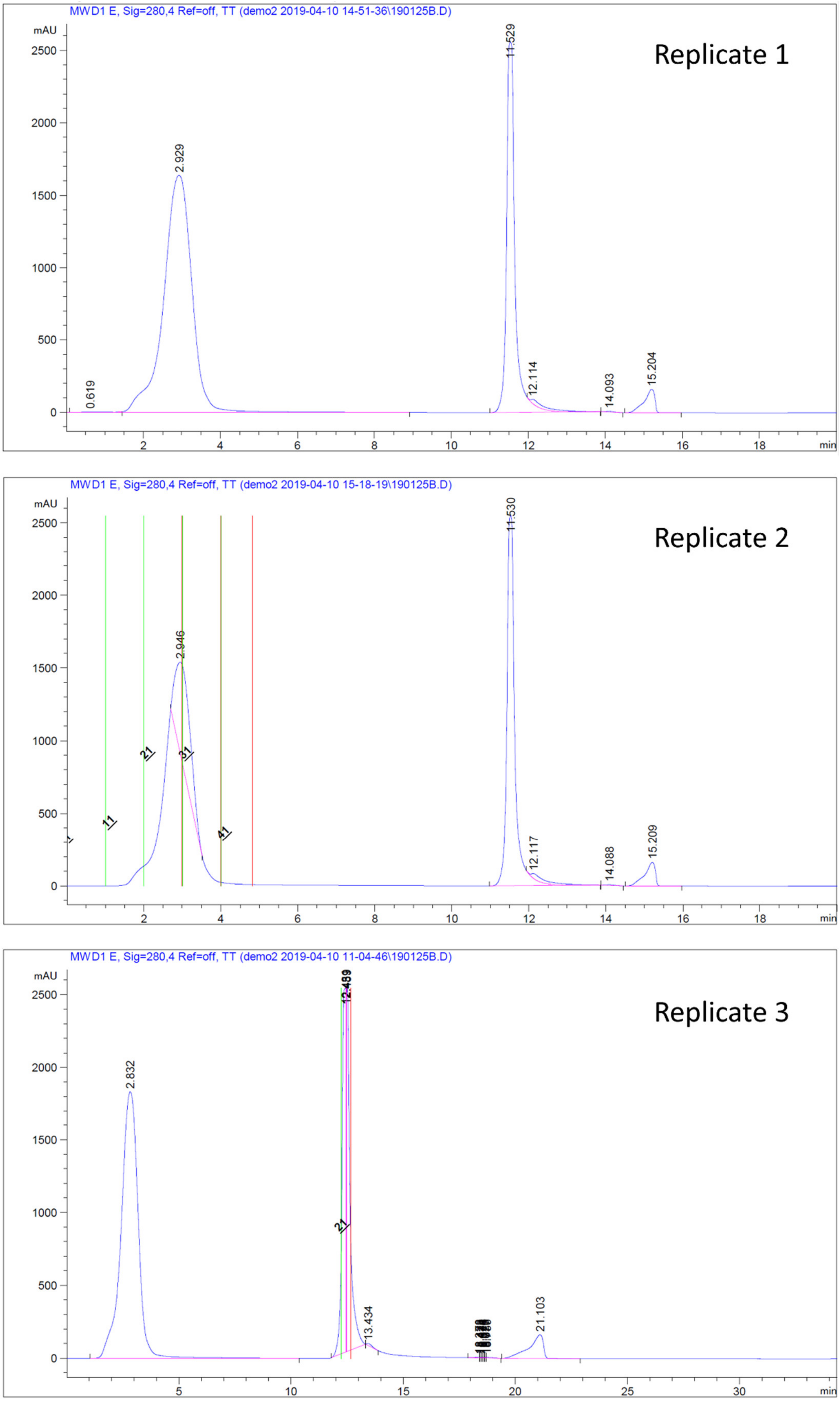

Figure S5A: Chromatograms obtained from the depletion of the seven most abundant proteins from plasma sample using Multiaffinity Removal Column human-7 (4,6 x 50mm) (Agilent Technologies).

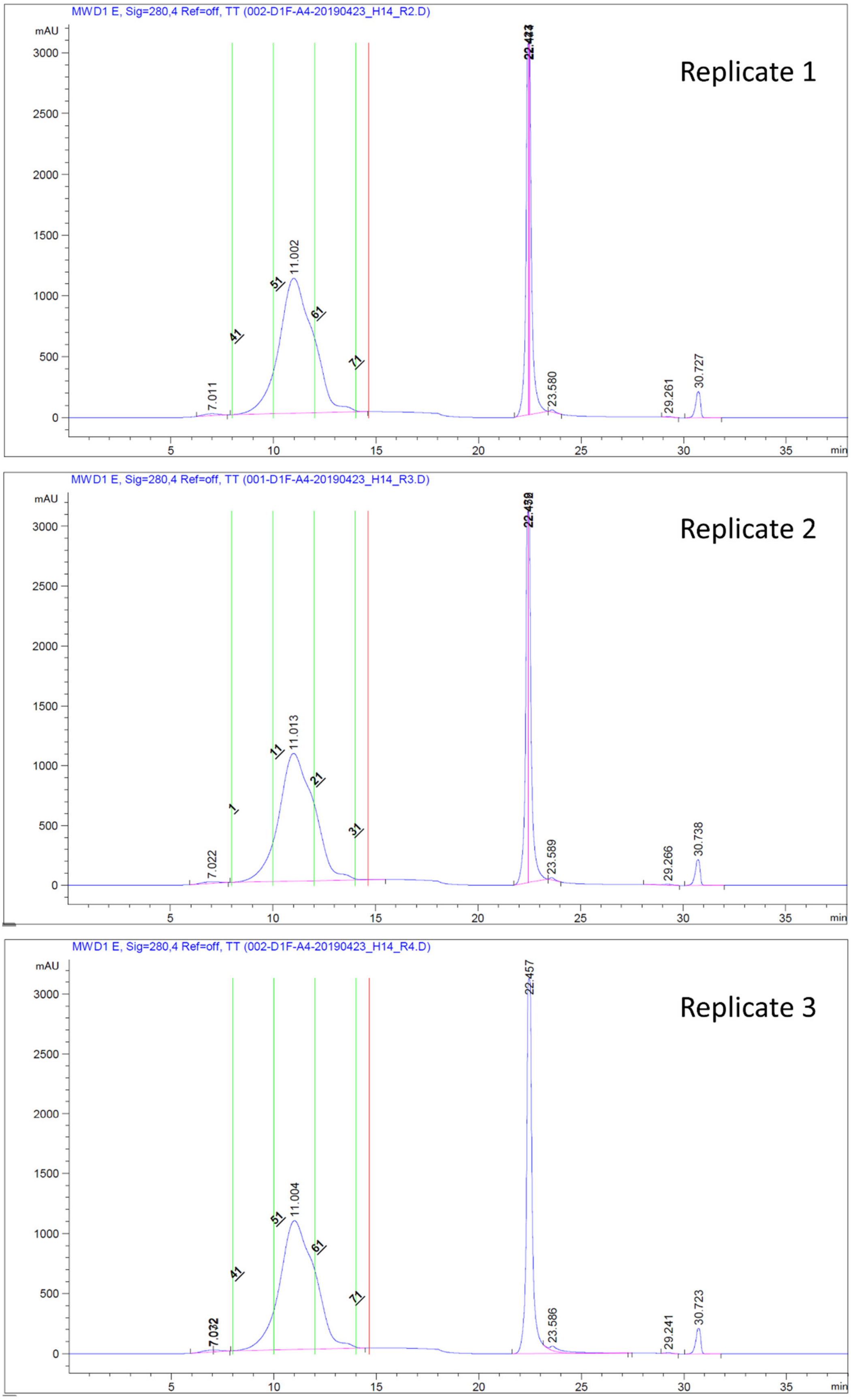

Figure S5B: Chromatograms obtained from the depletion of the fourteen most abundant proteins from plasma sample using Multiaffinity Removal Column human-14 (4,6 x 100mm) (Agilent Technologies).

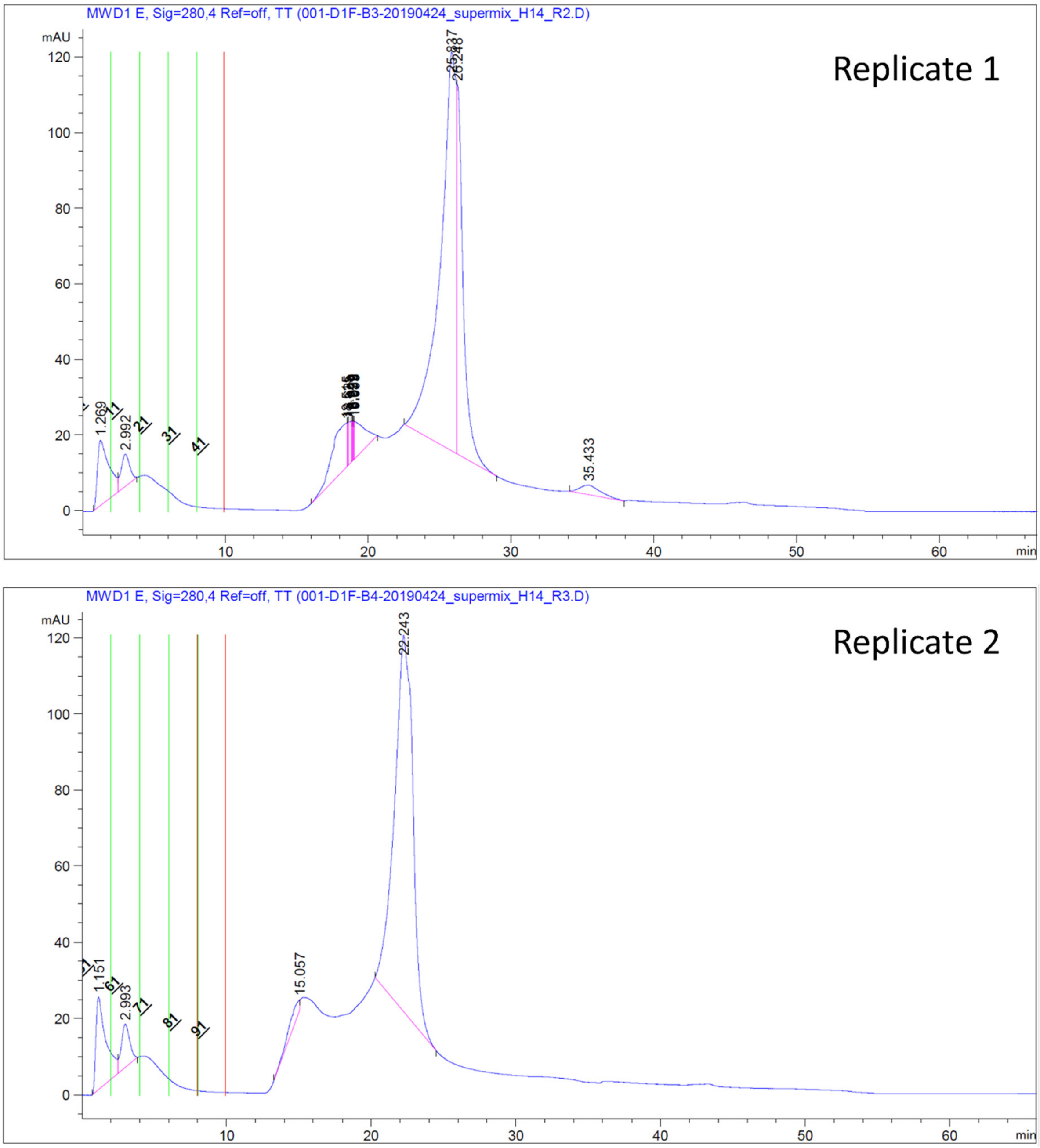

Figure S5C: Chromatograms obtained from the depletion of the approximately sixty most abundant proteins from plasma sample using Seppro® SuperMix LC2 column (6,4 x 63mm) (Sigma-Aldrich).

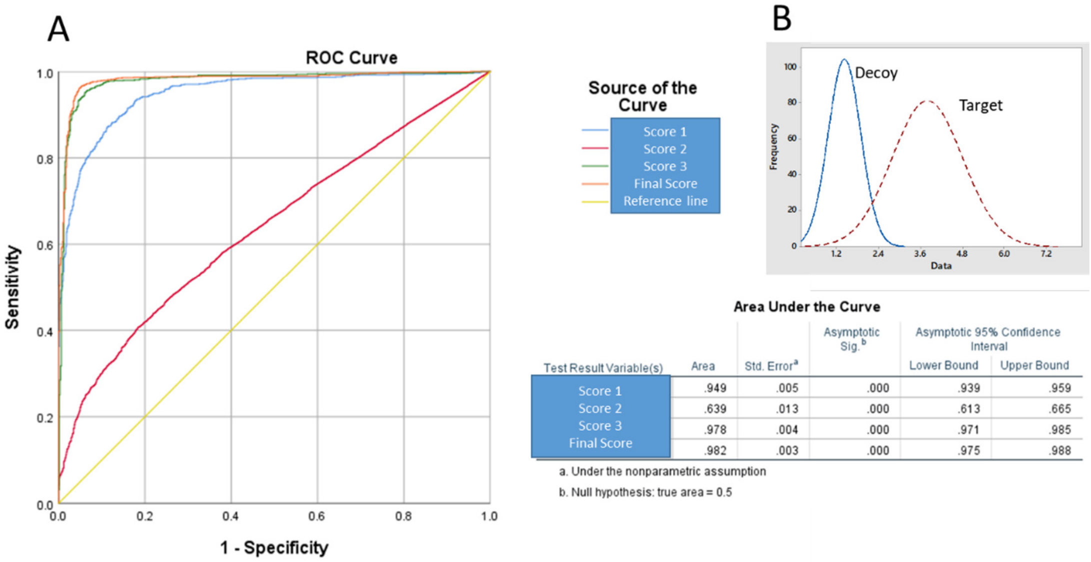

Area Under the Curve

Test Result Variable(s)	Area	Std. Error[a]	Asymptotic Sig.[b]	Asymptotic 95% Confidence Interval	
				Lower Bound	Upper Bound
Score 1	.949	.005	.000	.939	.959
Score 2	.639	.013	.000	.613	.665
Score 3	.978	.004	.000	.971	.985
Final Score	.982	.003	.000	.975	.988

a. Under the nonparametric assumption

b. Null hypothesis: true area = 0.5

Figure S6: Empirical Scores used for MS1 transferring evaluation. A) ROC curves of empirical scores based on the probabilities of plasma proteins to be identified by MS. The selected score (Final Score) represented by the orange line shows the best results for discriminating targets from decoy proteins. The final empirical score was created based on the protein intensity (Ii), the protein abundance rank (Proteins ordered by ascending MS intensities, Ranki), and the probability of being a plasma protein (PDi) based on customs databases: Final Score=(PDi*Ii)/Ranki. PD1 was set as 1 for proteins identified by MS2 in the undepleted sample. The table shows the ROC-AUC values and its significances after testing several scores B) Final score distribution for Target and Decoy proteins.
